# Secreted Glycoside Hydrolase Proteins as Effectors and Invasion Patterns of Plant-Associated Fungi and Oomycetes

**DOI:** 10.3389/fpls.2022.853106

**Published:** 2022-03-10

**Authors:** Ellie L. Bradley, Bilal Ökmen, Gunther Doehlemann, Bernard Henrissat, Rosie E. Bradshaw, Carl H. Mesarich

**Affiliations:** ^1^Bioprotection Aotearoa, School of Agriculture and Environment, Massey University, Palmerston North, New Zealand; ^2^Institute for Plant Sciences and Cluster of Excellence on Plant Sciences (CEPLAS), University of Cologne, Cologne, Germany; ^3^Department of Microbial Interactions, IMIT/ZMBP, University of Tübingen, Tübingen, Germany; ^4^DTU Bioengineering, Technical University of Denmark, Kongens Lyngby, Denmark; ^5^Architecture et Fonction des Macromolécules Biologiques (AFMB), UMR 7257 Centre National de la Recherche Scientifique (CNRS), Université Aix-Marseille, Marseille, France; ^6^Department of Biological Sciences, King Abdulaziz University, Jeddah, Saudi Arabia; ^7^Bioprotection Aotearoa, School of Natural Sciences, Massey University, Palmerston North, New Zealand

**Keywords:** glycoside hydrolases, fungi, oomycetes, effector proteins, invasion patterns

## Abstract

During host colonization, plant-associated microbes, including fungi and oomycetes, deliver a collection of glycoside hydrolases (GHs) to their cell surfaces and surrounding extracellular environments. The number and type of GHs secreted by each organism is typically associated with their lifestyle or mode of nutrient acquisition. Secreted GHs of plant-associated fungi and oomycetes serve a number of different functions, with many of them acting as virulence factors (effectors) to promote microbial host colonization. Specific functions involve, for example, nutrient acquisition, the detoxification of antimicrobial compounds, the manipulation of plant microbiota, and the suppression or prevention of plant immune responses. In contrast, secreted GHs of plant-associated fungi and oomycetes can also activate the plant immune system, either by acting as microbe-associated molecular patterns (MAMPs), or through the release of damage-associated molecular patterns (DAMPs) as a consequence of their enzymatic activity. In this review, we highlight the critical roles that secreted GHs from plant-associated fungi and oomycetes play in plant–microbe interactions, provide an overview of existing knowledge gaps and summarize future directions.

## Introduction

### Plant–Microbe and Microbe–Microbe Interactions

Filamentous fungi and oomycetes have evolved as efficient colonizers of plants, utilizing multiple strategies to interact with their hosts. Many of these organisms primarily reside in the plant apoplast during at least the first stages of colonization (Rocafort et al., 2020). The apoplast comprises all extracellular matrices and compartments outside the plasma membrane of plant cells (Sattelmacher, 2001) and contains toxic compounds and hydrolytic enzymes that disrupt fungal and oomycete growth (Doehlemann and Hemetsberger, 2013). Cell surface-localized immune receptor proteins, termed pattern recognition receptors (PRRs), also monitor the extracellular space for molecular invasion patterns to activate the plant immune system (Cook et al., 2015; van der Burgh and Joosten, 2019). Here, PRRs recognize microbe- or damage-associated molecular patterns (MAMPs or DAMPs, respectively) to activate immune responses that further slow or halt fungal and oomycete growth. These responses include the production of defensive compounds and reactive oxygen species (ROS), the deposition of polysaccharides and proteins (e.g., lignin, callose, and hydroxyproline-rich glycoproteins that reinforce or strengthen plant cell walls and infection sites; Kuć, 1997) and, in some cases, a localized cell death response (Dickman and Fluhr, 2013). Of the invasion patterns, MAMPs typically comprise broadly conserved molecules, such as proteins, lipids, and polysaccharides of invading fungi or oomycetes, whereas DAMPs are made up of endogenous molecules, such as cytosolic proteins, peptides, nucleotides, amino acids, and polysaccharides that are released from the plant upon fungal or oomycete attack (Newman et al., 2013; Raaymakers and van den Ackerveken, 2016; Hou et al., 2019; Tanaka and Heil, 2021).

Outside of the apoplast, plant-associated fungi and oomycetes are exposed to exudates that may contain a cocktail of plant-derived antimicrobial compounds (Jacoby et al., 2020). Furthermore, at all locations of colonization, secondary metabolite compounds, hydrolytic enzymes, and other proteins may be produced by other co-inhabiting microbes with roles in microbial antagonism (e.g., Carrión et al., 2019; Snelders et al., 2020, 2021). It is no surprise then that plant-associated fungi and oomycetes must neutralize or suppress plant defenses, whether constitutive or induced, as well as the antimicrobial activities of co-inhabiting microbes, in order to colonize their hosts. For this purpose, plant-associated fungi and oomycetes deploy a collection of virulence factors, termed effectors. These effectors, many of which are proteinaceous, function outside the plant cell in locations such as the apoplast or inside the plant cell, where they are translocated into various cell compartments (Ökmen and Doehlemann, 2014; He et al., 2020; Rocafort et al., 2020). In some cases, these effectors can also act as MAMPs (Thomma et al., 2011) or generate DAMPs that are recognized by PRRs to activate the plant immune system.

### Plant and Microbial Cell Walls

Immediately outside the plant plasma membrane is the plant cell wall, which forms part of the apoplast. The plant cell wall is a complex structure that fulfils diverse cellular functions ranging from maintenance of structural integrity to regulation of plant development (Zhang et al., 2021a). In terms of composition, more than 90% of the plant cell wall is made up of carbohydrates, with cellulose, hemicelluloses, and pectic polysaccharides the main carbohydrate components (Popper et al., 2011; Kumar and Turner, 2015). Crucially, the plant cell wall also provides a protective barrier against abiotic stresses and invading microbes (Vaahtera et al., 2019; Rui and Dinneny, 2020). Indeed, many plant-associated fungi and oomycetes must first breach the plant cell wall in order to colonize their hosts (Bellincampi et al., 2014). Breakdown or hydrolysis of the plant cell wall does, however, risk the release of DAMPs (such as oligogalacturonides, mixed-linked glucans, xyloglucans, and cellulose-derived oligomers) that can then be recognized by plant PRRs to activate the plant immune system (Aziz et al., 2007; Brutus et al., 2010; Ferrari et al., 2013; Benedetti et al., 2015; de Azevedo Souza et al., 2017; Claverie et al., 2018; Mélida et al., 2018, 2020; Rebaque et al., 2021).

Like in plants, the cell walls of plant-associated fungi and oomycetes play a vital role in maintaining the structural integrity of the cell, as well as in regulating development. Although types, distributions and linkages of cell wall carbohydrates vary from species to species (e.g., Gow et al., 2017), the main ones in fungi are chitin (β-1,4-N-acetylglucosamine, GlcNAc; inner layer) and β-1,3/1,6-glucans (outer layer), while the most abundant carbohydrates in oomycetes are cross-linked cellulose, β-1,4- and β-1,3/1,6-glucans (Wanke et al., 2021). Research on fungi, in particular, has shown that the cell wall is dynamic, with ongoing remodeling required for the morphological differentiation of specialized infection structures *in planta*. Such remodeling is necessary to protect fungal cell wall carbohydrates against hydrolysis by plant-derived enzymes, as well as to prevent their detection by PRRs (El Gueddari et al., 2002; Fujikawa et al., 2009, 2012; Oliveira-Garcia and Deising, 2013, 2016; Becker et al., 2016; Noorifar et al., 2021). As might be expected, however, this remodelling also runs the risk of releasing MAMPs (e.g., chitin and β-glucan fragments) that activate the plant immune system (Miya et al., 2007; Shimizu et al., 2010; Cao et al., 2014; Sánchez-Vallet et al., 2015; Fesel and Zuccaro, 2016; Rovenich et al., 2016; Wanke et al., 2020, 2021).

### Glycoside Hydrolases of Plant-Associated Fungi and Oomycetes

Many plant- and microbe-derived hydrolytic enzymes are carbohydrate-active enzymes (CAZymes). CAZymes are involved in the breakdown, biosynthesis, or modification of glycosidic bonds present in carbohydrates and glycoconjugates. Based on sequence and structural similarity of their functional domains, CAZymes can be classified into six main classes: glycoside hydrolases (GHs), carbohydrate esterases (CEs), polysaccharide lyases (PLs), glycosyltransferases (GTs), auxiliary activity enzymes (AAs), and carbohydrate-binding modules (CBMs; Lombard et al., 2014; Drula et al., 2022).^1^

GH proteins represent the largest class of CAZymes and are involved in the hydrolysis and/or rearrangement of glycosidic bonds in glycoconjugates, oligo- and polysaccharides (Henrissat, 1991; Henrissat and Davies, 1997). The CAZy database describes 172 GH families, which are grouped into 18 different GH clans (GH-A to -R) based on sequence similarity (Henrissat and Davies, 1997). Although a classification system based on sequence similarity is a very powerful way to predict the enzymatic activity of a novel GH enzyme, many GH families are polyspecific, meaning that one GH family can comprise enzymes with different substrate specificities (Henrissat, 1991).

The number and type of secreted GH proteins produced by plant-associated fungi and oomycetes with different lifestyles is highly variable. In biotrophic pathogens, as well as plant-associated endophytic microorganisms, a relatively low number of GHs targeting the plant cell wall are produced during host penetration and colonization, which minimizes host damage (Hane et al., 2020). On the other hand, necrotrophs and hemibiotrophs display more aggressive strategies during host colonization. At the necrotrophic stage of colonization, these pathogens secrete a wide range and number of GHs to directly or indirectly break down plant cell walls, and thus host cells, to feed on dead tissue (Hane et al., 2020). This is reflected in the genomes of these organisms, where it is generally accepted that biotrophs contain relatively few genes encoding plant cell wall-degrading GHs compared to necrotrophs and hemibiotrophs (Hane et al., 2020). Comparative genome analyses have revealed that, regardless of phylogenetic distance, the number and diversity of GH-encoding genes present in fungal and oomycete genomes is associated with their lifestyle. Although there are exceptions, many necrotrophic and hemibiotrophic fungi and oomycetes have around 300 GH-encoding genes (Zerillo et al., 2013; Zhao et al., 2013), while biotrophic and endophytic (symbiotic) fungi and oomycetes often contain only around 100 GH-encoding genes (Zerillo et al., 2013; Zhao et al., 2013; Hane et al., 2020). In contrast to necrotrophs and hemibiotrophs, biotrophs and symbiotic fungi lack GH6 family members, which display endoglucanase and cellobiohydrolase activities that target cellulose in the plant cell wall (Martin et al., 2010; Couturier et al., 2012; Zhao et al., 2013). Although saprophytes are not associated with plant diseases, they have a similar number of GH-encoding genes to hemibiotrophs and necrotrophs, supporting their well-known capacity for biomass decomposition (Zerillo et al., 2013; Zhao et al., 2013). On the other hand, saprophytic yeasts (such as those in the Class *Saccharomycetes*) lack many GH families including GH1, GH6, GH10, GH11, GH30, and GH79, and possess even fewer GH-encoding genes than biotrophs (Zhao et al., 2013).

In addition to lifestyle, the diversity of GH families is also correlated with cell wall composition of the host plants. For example, dicot plants encode more pectin in their cell wall compared to monocot plants. Thus, dicot-specific pathogens tend to have more pectinases, including GH28, GH88, and GH105 families, than monocot-specific pathogens (Zhao et al., 2013). Since the plant cell wall-degrading GH repertoire of plant-associated fungi and oomycetes is strongly associated with infection strategy or lifestyle, Hane et al. (2020) have developed a CAZyme-Assisted Training And Sorting of -trophy (CATAStrophy) pipeline to predict the lifestyle of a microorganism.

**Figure 1:**
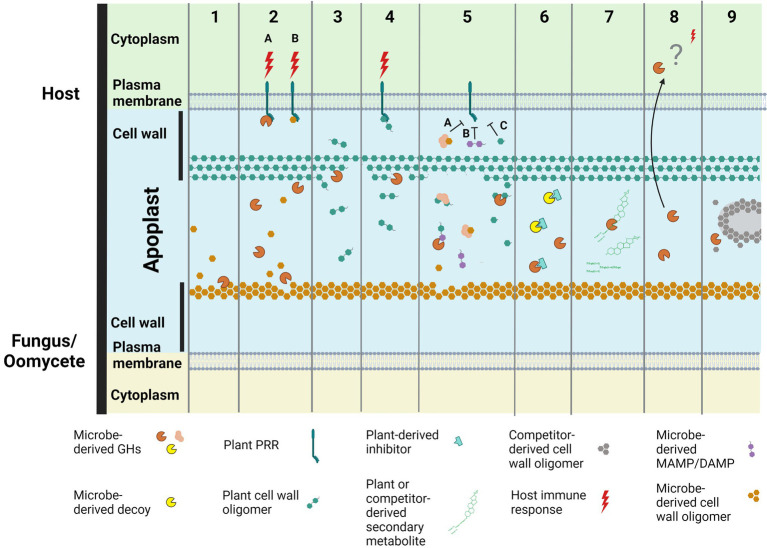
Secreted glycoside hydrolase (GH) proteins from plant-associated fungi and oomycetes play diverse roles in promoting plant colonization and/or activating plant immune responses. These roles include: **(1)** the modification of surface-associated carbohydrates present in their own cell walls to enable the remodeling of hyphal surfaces or infection structures produced during plant colonization; **(2)** the induction of plant immune responses, such as plant cell death, following their recognition as microbe-associated molecular patterns (MAMPs; **2A**), or the recognition of cell wall carbohydrate fragments (e.g., chitin or β-glucan oligomers) released from their own cell walls as a consequence of their activity [e.g. in **(1)**; **2B**], by pattern recognition receptors (PRRs) at the plant cell surface to provide plant resistance or susceptibility. Regarding the latter, plant cell death could, for example, result in a release of nutrients to support the growth of fungal or oomycete pathogens with a necrotrophic lifestyle, or drive a switch from biotrophy to necrotrophy for fungal or oomycete pathogens with a hemibiotrophic lifestyle; **(3)** nutrient acquisition through the release of carbohydrate fragments from plant cell walls or the breakdown of entire plant cells; **(4)** the induction of plant immune responses, such as cell death, following the recognition of plant cell wall carbohydrate fragments, generated as a consequence of their activity [e.g., in **(3)**] by PRRs at the plant cell surface to provide plant resistance or susceptibility. Again, plant cell death could support necrotrophy (as in **2B**); **(5)** the sequestration **(5A)**, modification **(5B)** or degradation **(5C)** of MAMPs or DAMPs to prevent their recognition by PRRs at the plant cell surface to prevent activation of plant immune responses; **(6)** acting as a decoy to bind host-produced proteins that would otherwise inhibit GH proteins produced by plant-associated fungi or oomycetes; **(7)** the detoxification of antimicrobial compounds produced by plants or microbial competitors (e.g., through the removal of a sugar group from enzymatic or non-enzymatic proteins or secondary metabolites); **(8)** functions that promote host colonization upon uptake into plant cells (currently uncharacterized); **(9)** manipulation of the host microbiome through, for example, breaking down the cell walls of microbial competitors. Figure created with BioRender.com.

Over recent years, it has become increasingly clear that secreted GH proteins of plant-associated fungi and oomycetes (i.e., those that are targeted extracellularly, but that lack a transmembrane domain or a glycophosphatidylinositol lipid modification site) play diverse roles in promoting host colonization and/or activating host immune responses (e.g., as effectors, MAMPs, or proteins that generate DAMPs; Kubicek et al., 2014; Rafiei et al., 2021; Figure 1; Supplementary Table 1). In this review, we highlight these roles, provide an overview of existing knowledge gaps and summarise future directions.

## Secreted GH Proteins From Plant-Associated Fungi and Oomycetes With Roles in Promoting Plant Colonization and/or Activating Plant Immune Responses

### Secreted Glycoside Hydrolase Family 3 (GH3) and 10 (GH10) Proteins

Saponins are fungi-toxic plant-derived secondary metabolites that play a role in plant defense, such as α-tomatine of tomato (*Solanum lycopersicum*) and avenacin of oat (*Avena sativa*; Bowyer et al., 1995). These saponins form a complex with sterols in the plasma membrane of fungi, but not oomycetes, resulting in a loss of membrane integrity (Bangham and Horne, 1962; Steel and Drysdale, 1988; Bowyer et al., 1995). Several fungal species, however, produce GH family 3 (GH3 tomatinase or avenacinase) or 10 (GH10 tomatinase) enzymes that break down or detoxify these saponins into non-toxic or less toxic compounds. For GH3 tomatinases, this is achieved through the removal of a terminal glucose from α-tomatine to give β_2_-tomatine, while for GH10 tomatinases, the entire lycotetraose moiety is removed to give tomatidine and β-lycotetraose (Turner, 1961; Osbourn, 1996).

Research on these enzymes was initially performed on a GH3 avenacinase from *Gaeumannomyces graminis* var. *avenae*, the necrotrophic fungal pathogen responsible for take-all disease in grasses. Similar to GH3 tomatinases, this avenacinase removes the terminal glucose molecules from avenacin A1 to give less toxic compounds (Crombie et al., 1986). Disruption of the avenacinase-encoding gene in *G. graminis* var. *avenae* resulted in an inability of the pathogen to cause disease on oat, suggesting an important role in pathogenicity (Bowyer et al., 1995).

Unlike the avenacinase-encoding gene from *G. graminis* var. *avenae*, targeted disruption of the GH3 tomatinase-encoding gene from *Septoria lycopersici*, a necrotrophic fungal pathogen responsible for leaf spot disease on tomato and other solanaceous plants, did not affect the ability of this pathogen to cause disease symptoms on currant tomato (*Solanum pimpinellifolium*; Martin-Hernandez et al., 2000). Instead, disruption only led to the increased expression of plant defense-related genes (Martin-Hernandez et al., 2000). Interestingly, while infection of *Nicotiana benthamiana* by wild type (WT) *S. lycopersici* resulted in clear disease lesions, no disease symptoms were observed upon infection with the tomatinase-deficient mutant (Bouarab et al., 2002). Disease symptoms were, however, observed when *N. benthamiana* leaves were pre-treated with either the GH3 tomatinase, or the resulting product (β_2_-tomatine), prior to infection with the tomatinase-deficient mutant (Bouarab et al., 2002). This suggested that the tomatinase enzyme of *S. lycopersici* is required for infection of *N. benthamiana* and, furthermore, that β_2_-tomatine is key to disease establishment by this pathogen (Bouarab et al., 2002).

Another tomatinase enzyme that has been studied in detail is FoTom1, a GH10 protein from *Fusarium oxysporum* f. sp. *lycopersici*, a hemibiotrophic fungal pathogen responsible for vascular wilt disease of tomato (Pareja-Jaime et al., 2008). In line with a role in virulence, ∆*fotom1* deletion mutants were significantly delayed in their ability to cause death of tomato plants, when compared to plants infected with WT *F. oxysporum* f. sp. *lycopersici* or a strain overexpressing *FoTom1* (Pareja-Jaime et al., 2008). It should be pointed out, however, that although a role in virulence was shown, ∆*fotom1* deletion mutants only showed 25% reduction in tomatinase activity in culture (Pareja-Jaime et al., 2008). Thus, it was anticipated that *F. oxysporum* f. sp. *lycopersici* produces other GH3 enzymes that also function as tomatinases (Pareja-Jaime et al., 2008).

In another example, *Cladosporium fulvum*, a biotrophic fungal pathogen responsible for tomato leaf mould disease, was shown to produce a functional GH10 tomatinase enzyme, CfTom1 (Ökmen et al., 2013). As with *FoTom1* (Pareja-Jaime et al., 2008), *CfTom1* expression was induced in culture in the presence of α-tomatine (Ökmen et al., 2013). During the early stages of tomato infection (3, 6, and 9 days post-inoculation, dpi), the expression of *CfTom1* was low, but was significantly induced at 12 dpi and peaked at 15 dpi (Ökmen et al., 2013). While both WT *C. fulvum* and ∆*cftom1* deletion mutants displayed similar levels of biomass during the initial penetration stages of infection, ∆*cftom1* biomass was significantly reduced from 10 dpi and, unlike WT infection, no accumulation of tomatidine was observed (Ökmen et al., 2013). Taken together, these results demonstrated that CfTom1 is required for the full virulence of *C. fulvum* on tomato (Ökmen et al., 2013).

### Secreted Glycoside Hydrolase Family 11 (GH11) Proteins

A large number of GH proteins from several GH families trigger cell death in host and/or non-host plants (Supplementary Table 1). In many cases, the ability of these proteins to trigger cell death is independent of enzymatic activity, suggesting that they are recognized as MAMPs by PRRs localized on the plant cell surface (Supplementary Table 1). One such example is ethylene-inducing xylanase (EIX), a GH family 11 (GH11) protein with β-1-4-endoglucanase activity from *Trichoderma viride*, the symbiotic biocontrol fungus associated with plant roots and soil (Fuchs and Anderson, 1987; Dean et al., 1989; Fuchs et al., 1989; Dean and Anderson, 1991). EIX (hereafter referred to as TvEIX) triggers a wide range of plant immune responses (mostly in *N. benthamiana* cv. Xanthi), including the induction of ethylene biosynthesis, electrolyte leakage, pathogenesis-related (PR) protein expression, phytoalexin and ROS production, as well as cell death (Bailey et al., 1990, 1992; Lotan and Fluhr, 1990; Fluhr et al., 1991; Avni et al., 1994; Yano et al., 1998; Ron et al., 2000; Laxalt et al., 2007). Consistent with the recognition of TvEIX as a MAMP, mutation of the active site residues in this protein revealed that the xylanase activity of TvEIX is not required for cell death elicitation (Furman-Matarasso et al., 1999). This is in line with a previous study which concluded, based on protoplast assays, that the enzymatic activity of TvEIX is also not required for induction of ethylene biosynthesis (Sharon et al., 1993). It has since been shown that a five-amino acid surface-exposed pentapeptide motif, TKLGE, which is not required for enzymatic activity, is the epitope recognized by *N. benthamiana* cv. Xanthi (Rotblat et al., 2002).

The cell death responses triggered by TvEIX in *N. benthamiana* cv. Xanthi and tomato are governed by a single dominant locus (Bailey et al., 1993; Ron et al., 2000). In tomato, this locus is made up of two genes, *SlEIX1* and *SlEIX2*, which both encode leucine-rich repeat receptor-like protein (LRR-RLP) PRRs capable of binding TvEIX (Ron and Avni, 2004). Of these two PRRs, however, only SlEIX2 is capable of initiating immune responses upon recognition of TvEIX, while SlEIX1 instead acts as a decoy immune receptor to attenuate TvEIX-induced immune signaling by SlEIX2 (Ron and Avni, 2004; Bar et al., 2010). It is known that LRR-RLPs often dimerize with receptor-like kinases (RLKs), such as suppressor of BIR1 (SOBIR1) and/or BRI1-associated kinase-1 (BAK1), in order to initiate signal transduction (Li et al., 2002; Liebrand et al., 2013, 2014). Interestingly, while SlEIX2 associates with the co-receptor SOBIR1 (Liebrand et al., 2013), the co-receptor BAK1 has been shown to interact with SlEIX1 only, indicating that the recognition of TvEIX by SlEIX2 is BAK1-independent (Bar et al., 2010). Based on these and other experiments, a model was put forward in which SlEIX1, in the presence of BAK1, binds to TvEIX, and heterodimerizes with SlEIX2 to prevent SlEIX2 endocytosis and resultant plant immune responses (Bar and Avni, 2009; Bar et al., 2010, 2011). Because longer TvEIX exposure leads to a stronger immune response, including cell death (Bar et al., 2010), it is anticipated that the function of SlEIX1 is to prevent immune responses over the short term which, in turn, enables symbiotic *T. viride* to enter host plants. Importantly, this function is not expected to prevent immune responses from occurring against pathogenic microbes when necessary (Bar et al., 2010, 2011).

Following on from the research on TvEIX and SlEIX1/SlEIX2, an LRR-RLP PRR named NbEIX2, which is orthologous to SlEIX2 from tomato, was identified in *N. benthamiana* (Yin et al., 2021). NbEIX2 recognizes VdEIX3, a TvEIX-like protein from *Verticillium dahliae*, a broad host-range, hemibiotrophic fungal pathogen responsible for vascular wilt disease (Yin et al., 2021). While NbEIX2 constitutively associates with both BAK1 and SOBIR1, co-immunoprecipitation assays revealed that NbEIX2 dissociates from BAK1 after treatment with VdEIX3, indicating that the cell death and other immune responses elicited by VdEIX3 (i.e., ROS production and the induction of *PR* and MAMP-triggered defense genes) are BAK1-independent (Yin et al., 2021). This result was corroborated by gene silencing experiments in which VdEIX3 triggered cell death and other immune responses, including ROS production, in *N. benthamiana* plants silenced for the *BAK1* gene (Yin et al., 2021). The same was observed in *N. benthamiana* plants silenced for *SOBIR1*, indicating that the immune responses triggered by VdEIX3 are also SOBIR1-independent (Yin et al., 2021).

Another well characterized fungal GH11 is BcXyn11A, a protein with β-1,4-endoxylanase activity from *Botrytis cinerea*, the broad host-range necrotrophic pathogen responsible for grey mould disease (Brito et al., 2006). During invasion of tomato, the *BcXyn11A* gene is expressed from the beginning of the infection process, increasing from 24 to 48 h post-inoculation (hpi), with Δ*bcxyn11a* deletion mutants showing significantly reduced virulence (Brito et al., 2006). BcXyn11A hydrolyses the linear backbone of xylan, which is the main hemicellulose component of plant cell walls. The BcXyn11A protein was shown to induce immune responses including the upregulation of defense-related genes, ROS production, electrolyte leakage, and cell death, when infiltrated into leaves of tomato and *Nicotiana tabacum* cvs. Havana, Alcalá and Paraíso (Brito et al., 2006; Noda et al., 2010; Frías et al., 2019). Like TvEIX, enzymatic activity is not required for cell death induction, suggesting that BcXyn11A is recognized as a MAMP by host plants (Noda et al., 2010). Remarkably, although Δ*bcxyn11a* deletion mutants had reduced virulence on both tomato leaves and grape berries (Brito et al., 2006), when Δ*bcxyn11a* mutants were complemented with copies of the *BcXyn11A* gene that encode enzymatically inactive versions of the protein, virulence was restored, suggesting that BcXyn11A contributes to the virulence of *B. cinerea* through cell death induction (i.e., necrosis), rather than by enzymatic activity (Noda et al., 2010).

A 25-amino acid peptide, Xyn25, has since been identified as the component of BcXyn11A that is sufficient for elicitation of cell death, as well as other immune responses, in *N. tabacum* cv. Havana and *S. lycopersicum* cv. Moneymaker (Frías et al., 2019). From this peptide, two regions consisting of four consecutive amino acid residues (YGWT and YYIV, respectively) are required for the induction of defense responses (Frías et al., 2019). These amino acids are partially exposed on the predicted tertiary structure of BcXyn11A and are conserved across other xylanases like TvEIX (Frías et al., 2019). Interestingly, a xylanase inhibitor protein, TAXI-I, has been identified from wheat (*Triticum aestivum*) that can prevent the necrotizing activity of BcXyn11A when expressed in the host plant *Arabidopsis thaliana* (Brutus et al., 2005; Tundo et al., 2020). As the 25-amino acid cell death elicitation region is located next to the catalytic site in the predicted tertiary structure of BcXyn11A, it is hypothesized that binding of TAXI-I to the catalytic site hides the necrotizing region from recognition by a putative PRR (Tundo et al., 2020).

### Secreted Glycoside Hydrolase Family 12 (GH12) Proteins

Like the GH11 proteins described above, a diverse range of GH family 12 (GH12) proteins belonging to both plant-associated oomycetes and fungi are capable of triggering cell death when transiently expressed in, or infiltrated into, plants (Supplementary Table 1). One of the best studied is PsXEG1, a GH12 protein with hydrolytic activity towards both β-glucan and xyloglucan that was identified from *Phytophthora sojae*, a hemibiotrophic oomycete pathogen (Ma et al., 2015b). PsXEG1 triggers cell death in some solanaceous plant species, including the model host *N. benthamiana*, as well as the native fabaceous host, soybean (*Glycine max*; Ma et al., 2015b). While either silencing of *PsXEG1*, or mutation of the catalytic residues present in the protein it encodes, significantly reduced *P. sojae* virulence on soybean, PsXEG1 catalytic mutants were still capable of eliciting cell death. This suggested that not only is PsXEG1 an important virulence factor of *P. sojae* but also that its contribution to virulence on soybean is dependent on enzyme activity. Furthermore, given that enzymatic activity is not required for cell death induction by PsXEG1, these results suggested that PsXEG1 is recognized as a MAMP (Ma et al., 2015b, 2017).

PsXEG1 is secreted into the apoplast as two isoforms. The larger isoform is N-glycosylated at positions N174 and N190, protecting it from degradation by the apoplastic aspartic protease of soybean, GmAP5. However, the smaller, non-glycosylated isoform of PsXEG1 is bound by GmAP5 and quickly degraded (Ma et al., 2015b; Xia et al., 2020). In the apoplast, non-glycosylated PsXEG1 is also bound by the soybean glucanase inhibitor protein, GmGIP1, which not only prevents xyloglucan hydrolysis by this isoform of PsXEG1 but also inhibits the contribution of this isoform to *P. sojae* virulence (Ma et al., 2017; Xia et al., 2020). Interestingly, only one other GH12 protein of *P. sojae*, PsXLP1, which shares 67% amino acid identity and a similar expression profile with PsXEG1 (i.e., highly expressed from 20 min to 2 h during *P. sojae* infection of soybean; Ma et al., 2015, 2017), was found to bind to GmGIP1 (Ma et al., 2017). PsXLP1 harbours a C-terminal deletion that results in the loss of E222, one of the residues essential for enzyme activity (Ma et al., 2015b), rendering it catalytically inactive (Ma et al., 2017). Despite this C-terminal deletion, PsXLP1 was found to bind GmGIP1 with five times higher affinity than PsXEG1 (Ma et al., 2017). In doing so, PsXLP1 acts as a decoy, preventing GmGIP1 inhibition of PsXEG1 (Ma et al., 2017).

Using a virus-induced-gene silencing (VIGS) approach, Wang et al. (2018) showed that the recognition of PsXEG1 as a MAMP in the apoplast of *N. benthamiana* is mediated by the LRR-RLP PRR RXEG1, which is an ortholog of the SlEIX2 and NbEIX2 PRRs described above. Notably, RXEG1 recognizes not only PsXEG1 but also a broad range of GH12 cell death elicitors from both fungal and oomycete species (Wang et al., 2018). This recognition is achieved through the LRR domain of the PRR, with co-operation of both BAK1 and SOBIR1 (Ma et al., 2015b; Wang et al., 2018). Here, both co-receptors interact with RXEG1 even in the absence of PsXEG1, although interaction between RXEG1 and BAK1 is enhanced in the presence of PsXEG1 (Wang et al., 2018).

GH12 proteins have also been characterized in *V. dahliae*. Of the six GH12 proteins identified in this species by Gui et al. (2017), only two (VdEG1 and VdEG3) triggered plant immune responses (ROS accumulation, callose deposition, and cell death) in *N. benthamiana*. These responses were independent of their cellulolytic activity (Gui et al., 2017). Similar to PsXEG1, VdEG1-triggered cell death was dependent on both BAK1 and SOBIR1, while only BAK1 was required for VdEG3-triggered cell death (Gui et al., 2017). A deletion analysis demonstrated that while full length VdEG1 was required for induction of cell death, only a 63-amino acid peptide from VdEG3 was necessary (Gui et al., 2017). Interestingly, VdEG3 is composed of both a GH12 domain and a carbohydrate-binding module family 1 (CBM1) domain, but expression of the VdEG3 GH12 domain alone triggered stronger cell death than full length VdEG3. This suggested that the CBM1 domain suppresses VdEG3-GH12-triggered cell death (Gui et al., 2017). The addition of more CBM1 domains appeared to have an additive effect, suppressing both VdEG3-triggered cell death and ROS accumulation (Gui et al., 2017). Furthermore, the VdEG3-CBM domain also suppressed cell death triggered by GH12 proteins from other fungal species (Gui et al., 2017). Of note, several other microbial GH proteins have been found to contain CBM domains (Tundo et al., 2021; Takeda et al., 2022). These include MoCel10A and MoCel6A, a GH10 xylanase and GH6 cellobiohydrolase, respectively, from *Magnaporthe oryzae*, the hemibiotrophic fungal pathogen responsible for blast disease of rice (*Oryza sativa*). Recently it has been shown that both of these proteins interact through their CBM domains with the rice protein OsCBMIP, which subsequently inhibits their plant cell wall degrading activity (Takeda et al., 2022).

Infection of *N. benthamiana* with *V. dahliae* strains harboring a deletion of either the *VdEG1* or *VdEG3* gene resulted in increased virulence and significantly increased fungal biomass compared to infection by WT *V. dahliae* (Gui et al., 2017). However, the opposite was true during infection of cotton (*Gossypium hirsutum*). In this case, there was a reduction in both *Verticillium* wilt symptoms and fungal biomass. During infection of cotton with *V. dahliae VdEG1* or *VdEG3* complementation strains, fungal biomass was restored to WT levels as expected; however, complementation with catalytic mutant versions of these genes did not restore biomass, suggesting that enzyme activity is required for the virulence function of VdEG1 and VdEG3 in this plant host. In addition, VdEG1 and VdEG3 were unable to trigger plant cell death or ROS accumulation, suggesting they are not recognised as MAMPs by cotton (Gui et al., 2017).

Another example of a secreted GH12 protein that is recognized as a MAMP by plants is FoEG1 from *F. oxysporum* (Zhang et al., 2021d). During infection of cotton and tomato roots, *FoEG1* is most highly expressed during the early stages of infection, from 24 to 48 hpi (Zhang et al., 2021d). Deletion mutants (Δ*foeg1*) demonstrated reduced virulence on cotton plants (Zhang et al., 2021d). This reduction in virulence was also observed for Δ*foeg1* mutants complemented with a catalytically-inactivated version of the gene, indicating that the enzymatic activity of FoEG1 is required for full virulence of the pathogen (Zhang et al., 2021d). In line with the protein being recognized as a MAMP, FoEG1 triggered cell death, independent of enzymatic activity, upon infiltration into *N. benthamiana*, *N. tabacum*, tomato and cotton and, in *N. benthamiana*, was dependent on both BAK1 and SOBIR1 (Zhang et al., 2021d). Other defense responses reported in *N. benthamiana* included ROS accumulation, callose deposition and the induction of defense-related genes (Zhang et al., 2021d). Notably, an internal 86-amino acid fragment from amino acid positions 144–229 of FoEG1 was found to be sufficient for cell death induction and ROS accumulation in *N. benthamiana* (Zhang et al., 2021d).

Secreted GH12 proteins from plant-associated fungi and oomycetes also play an active role in DAMP release. Examples include MoCel12A and MoCel12B, two secreted GH12 proteins with β-glucanase (endoglucanase) activity from *M. oryzae* (Takeda et al., 2010; Yang et al., 2021). The expression of both *MoCel12A* and *MoCel12B* is upregulated during the early stages of infection when the fungus is undergoing biotrophic growth. Here, expression is initiated at 8 h post-inoculation (hpi), around the time of primary infection hyphae formation, then peaks at 24 and 12 hpi, respectively (Yang et al., 2021). While *∆mocel12a* deletion mutants did not display reduced virulence on rice, *∆mocel12a/b* double-mutants exhibited enhanced virulence, as measured by more severe disease symptoms and increased fungal biomass (Yang et al., 2021). Furthermore, strains overexpressing *MoCel12A* had reduced biomass during host infection (Yang et al., 2021). Taken together, these results suggested that MoCel12A and/or MoCel12B negatively contribute to the virulence of *M. oryzae*. This is supported by the finding that the ectopic expression of *MoCel12A* in rice leads to enhanced resistance against this fungus, associated with the significant upregulation of immune-responsive genes, a dwarf phenotype, and the formation of spontaneous lesions on leaves of transgenic plants (Yang et al., 2021).

Notably, ectopic expression of an enzymatically inactive variant of MoCel12A, with mutations in both active site residues, failed to provide resistance to *M. oryzae*, suggesting that MoCel12A activates the plant immune system through the production of DAMPs (Yang et al., 2021). In line with this, only extracts from rice cell walls pre-incubated with active MoCel12A or MoCel12B enzymes triggered a ROS burst and induction of immunity-related genes in rice suspension cells (Yang et al., 2021). It was subsequently established that MoCel12A and MoCel12B release two major Poaceae-specific oligosaccharides from the hemicellulose component of rice cell walls, namely the trisaccharide 3^1^-β-D-cellobiosyl-glucose (BGTRIB) and the tetrasaccharide 3^1^-β-D-cellotriosyl-glucose (BGTETB), which are detectable along with MoCel12A in the apoplastic wash fluid of *M. oryzae*-infected rice plants, and that activate the rice immune system (Yang et al., 2021). Consistent with this finding, BGTRIB, as well as another immune system-activating oligosaccharide identified in the study, 3^3^-β-D-glucosyl-cellotriose (BGTETC), can prime the immune system of rice, with BGTRIB and BGTETC pre-treatment providing enhanced resistance against rice blast disease (Yang et al., 2021). Yang et al. (2021) also showed that the abovementioned oligosaccharides are perceived by the PRR OsCERK1, a lysin motif (LysM) RLK, but not the LysM-RLP PRR OsCEBiP (Yang et al., 2021). This recognition induces OsCERK1 homodimerization, as well as heterodimerization with OsCEBiP, which Yang et al. (2021) suggest likely form OsCERK1-OsCEBiP tetramers to transduce immune signaling.

In the case of MoCel12A and MoCel12B, it remains unclear what their primary role is in promoting host colonization. However, it has been shown that the virulence of *M. oryzae* is enhanced when *MoCel12A* is overexpressed in an *oscerk1* mutant background, suggesting that the endoglucanase activity of this protein is important for infection when released oligosaccharides cannot be perceived (Yang et al., 2021). As such, MoCel12A and MoCel12B may play a vital role in nutrient acquisition but, through this activity, can produce DAMPs that are inadvertently recognized by OsCERK1 (Yang et al., 2021). It should be noted, though, that roles in the modification of MAMPs or other DAMPs, or in the switch from biotrophy to necrotrophy, have not yet been ruled out.

### Secreted Glycoside Hydrolase Family 16 (GH16) Proteins

Although nothing has yet been shown for plant-associated oomycetes, research is emerging that selected secreted GH proteins from plant-associated fungi can be translocated into host cells. One such example is BcCrh1, a GH family 16 (GH16) transglycosylase from *B. cinerea* that requires dimerization for enzymatic activity (Bi et al., 2021). BcCrh1 was originally identified in the secretome of *B. cinerea*-infected bean (*Phaseolus vulgaris*) leaves (Zhu et al., 2017) and triggers cell death and other defense responses (e.g., ROS accumulation, callose deposition and the upregulation of defense-related genes) in *N. benthamiana* and tomato (Bi et al., 2021). During host infection, the expression of *BcCrh1* is induced following first contact of the fungus with the plant, and peaks at 12 hpi, while at the protein level, BcCrh1 is initially released into the apoplast from structures called infection cushions (Bi et al., 2021). Mutation of catalytic site residues (E120Q/D122H/E124Q) demonstrated that the ability of BcCrh1 to trigger cell death was independent of enzymatic activity (Bi et al., 2021). However, this cell death could still be triggered by a mutant version of the protein incapable of forming dimers (Bi et al., 2021).

Notably, following secretion into the apoplast of *N. benthamiana*, BcCrh1 was shown to be targeted to the cell cytoplasm, with a version of the protein lacking a signal peptide (i.e., confined to the plant cytoplasm) retaining its ability to trigger cell death (Bi et al., 2021). A 35-amino acid region of BcCrh1 (position 93–127) was determined to be sufficient for this cell death-inducing activity, while a 53-amino acid region (position 21–74, directly after the native signal peptide) was found to mediate the uptake into plant cell cytoplasm (Bi et al., 2021). Interestingly, deletion or overexpression of *BcCrh1* had no effect on *B. cinerea* virulence (Bi et al., 2021). However, overexpression of the enzyme-inactive version of BcCrh1 in a ∆*BcCrh1* mutant background significantly reduced *B. cinerea* virulence (Bi et al., 2021). This reduction is thought to be due, in part, to impaired infection cushion formation, as a result of accumulating enzyme-inactive dimers (Bi et al., 2021). Taken together, it has been proposed that BcCrh1 may have a role in the formation of infection cushions, and that excessive BcCrh1 is released from these structures to induce plant cell death (i.e., as an effector) upon translocation into host cells from the apoplast (Bi et al., 2021).

### Secreted Glycoside Hydrolase Family 17 (GH17) Proteins

Another example of a secreted GH protein that releases a DAMP through its enzymatic activity is CfGH17-1, an apoplastic GH family 17 (GH17) protein with 1,3-β-glucanase activity from *C. fulvum* (Ökmen et al., 2019). *CfGH17-1* expression is downregulated during the biotrophic phase of *C. fulvum* growth, but upregulated during later stages of infection when tomato leaves are necrotic and the fungus is saprophytic (Ökmen et al., 2019). CfGH17-1 was shown to trigger cell death in three out of four lines of Moneymaker tomato tested, but not in the non-host plants *N. benthamiana* and *N. tabacum* (Ökmen et al., 2019). Through targeted mutation of the enzymatic active site residues present in CfGH17-1, cell death activity could be prevented, indicating that the protein is not recognized as a MAMP (Ökmen et al., 2019). Instead, it is anticipated that CfGH17-1 releases a sugar molecule from the plant cell wall that is subsequently recognized as a DAMP by an uncharacterized PRR present in specific tomato lines, but not in *N. benthamiana* or *N. tabacum* (Ökmen et al., 2019). Based on this observation, it was proposed that CfGH17-1 likely plays a role in nutrient acquisition by acquiring sugar molecules from the host cell wall to support the growth and reproduction of *C. fulvum* during the late stages of infection when the host is no longer able to recognize and respond to DAMPs (Ökmen et al., 2019). Consistent with this, symptom development remained unchanged in tomato plants infected with Δ*cfgh17-1* deletion mutants of *C. fulvum*, when compared to plants infected with WT fungus, whereas fewer disease symptoms were observed in tomato plants infected with strains constitutively overexpressing the *CfGH17-1* gene (Ökmen et al., 2019).

### Secreted Glycoside Hydrolase Family 18 (GH18) Proteins

Although the manipulation of chitin-triggered immunity in plants by pathogen effectors is not new, the involvement of GH proteins in this process has only recently been shown. A notable example is MpChi, an enzymatically inactive, secreted chitinase-like GH18 protein from *Moniliophthora pernicosa*, the hemibiotrophic fungal pathogen responsible for witches’ broom disease of cacao (*Theobroma cacao*; Fiorin et al., 2018). MpChi, which is encoded by a gene that is highly expressed during biotrophic infection of cacao, is able to bind chitin oligomers (Fiorin et al., 2018). However, it was determined that MpChi harbours an amino acid substitution in the catalytic motif conserved in GH18 chitinases (E167Q), as well as substitution of a residue that forms part of the catalytic pocket in these enzymes (M238L); together these substitutions abolish chitinolytic activity (Fiorin et al., 2018). Strikingly, in line with a role in manipulating chitin-triggered immunity, the treatment of *N. tabacum* cell suspensions with MpChi prevented defense gene expression and medium alkalinization that would otherwise be triggered by chitin oligomers (Fiorin et al., 2018). As this role was dependent on the chitin binding capacity of MpChi, it was determined that MpChi prevents chitin-triggered immunity through the sequestration of immunogenic chitin fragments (Fiorin et al., 2018).

Interestingly, the orthologue of MpChi from *Moniliophthora roreri*, a related hemibiotrophic pathogen of cacao responsible for frosty pod rot disease, has canonical catalytic residues and is enzymatically active (Fiorin et al., 2018). However, a paralogous secreted GH18 protein from this pathogen, MrChi, was identified that has a different amino acid substitution in its GH18 chitinase catalytic motif (D135N; Fiorin et al., 2018). This substitution resulted in reduced, but not abolished, enzymatic activity (Fiorin et al., 2018). Like MpChi, MrChi is encoded by a gene that is highly expressed during biotrophic infection and can suppress the chitin-triggered immune response in *N. tabacum* cell suspensions (Fiorin et al., 2018). Taken together, this study highlighted that GH18 proteins from two cacao pathogens of the same genus have independently evolved to prevent chitin-triggered immunity through the sequestration of immunogenic chitin fragments (Fiorin et al., 2018).

Investigations have also been led into the roles of enzymatically active GH18 chitinases from fungal pathogens in modulating chitin-triggered immunity. In 2019, two separate studies focused on the same extracellularly-targeted GH18 chitinase from *M. oryzae*, named MoChia1 by Yang et al. (2019) and MoChi by Han et al. (2019). *MoChia1*/*MoChi* (hereafter referred to as *MoChia1*) is highly expressed at 48 hpi in rice (Yang et al., 2019). Like many other GH proteins described above, MoChia1 is recognized as a MAMP and can induce a ROS burst and callose deposition in rice cell suspensions independent of enzymatic activity (Yang et al., 2019). In line with the recognition of MoChia1 as a MAMP, overexpression of *MoChia1* in rice resulted in reduced virulence (Yang et al., 2019). Interestingly, deletion of *MoChia1* in *M. oryzae* gave delayed appressorium and germ-tube formation on glass coverslips (Yang et al., 2019), as well as a slower post-penetration growth-rate, fewer lesions, and reduced biomass in rice leaves (Han et al., 2019; Yang et al., 2019). Further analyses revealed that these *in planta* phenotypes were at least partially mediated by an enhanced immune response, as measured by the increased expression of defense-related genes in rice, suggesting that MoChia1 also plays a role in the suppression of MAMP-triggered immunity (Han et al., 2019; Yang et al., 2019).

Both studies also identified an interacting partner of MoChia1 in rice. More specifically, Yang et al. (2019) identified an interaction between the carbohydrate-binding domain of MoChia1 and the plasma membrane-localized tetratricopeptide-repeat protein OsTPR1, while Han et al. (2019) identified an interaction between MoChia1 and the plasma membrane-localized, chitin-binding, jacalin-related lectin OsMBL1. In both cases, the genes that encode these proteins were induced by *M. oryzae* infection, suggesting a role in plant defense (Han et al., 2019; Yang et al., 2019). In support of this, overexpression of *OsTPR1* or *OsMBL1* in rice resulted in fewer lesions by *M. oryzae* and was concomitant with a significant reduction in fungal biomass, as well as the activation of defense-related genes (Han et al., 2019; Yang et al., 2019).

More in-depth analyses determined that MoChia1 suppresses the chitin-triggered ROS burst in rice (Han et al., 2019; Yang et al., 2019), but that this suppression can be prevented by OsTPR1 (Yang et al., 2019). Coincident with this, MoChia1 suppressed the chitin-induced ROS burst in rice plants overexpressing *OsTPR1* (Yang et al., 2019). The immune response following the recognition of MoChia1 as a MAMP, however, was not suppressed upon *OsTPR1* overexpression (Yang et al., 2019). Yang et al. (2019) discovered that, although OsTPR1 was unable to bind chitin, the interaction between OsTPR1 and MoChia1 was stronger than the interaction between MoChia1 and chitin. Based on these and other results, it was proposed that, through its interaction with MoChia1, OsTPR1 allows free chitin that would otherwise be bound or degraded by MoChia1 to activate chitin-triggered immune responses (Yang et al., 2019).

Unlike OsTPR1, Han et al. (2019) demonstrated that OsMBL1 interacts with chitin and observed a negative correlation between the amount of MoChi1 present and the amount of chitin bound by OsMBL1. Thus, it was proposed that OsMBL1 is a cell surface-localized PRR required for the recognition of chitin oligomers in rice, and that MoChia1 and OsMBL1 compete with each other for the binding of these chitin oligomers (Han et al., 2019). As MoChia1 has a higher affinity for chitin oligomers than OsMBL1, MoChia1 can then degrade or sequester the chitin oligomers to prevent their recognition by OsMBL1, and in doing so, prevent the activation of chitin-triggered immune responses (Han et al., 2019).

In addition to the functions described above, it is also expected that a subset of GH18 proteins secreted by plant-associated fungi and oomycetes during host colonization function as effectors with roles in manipulating plant microbiota. Mycoparasite-produced chitinases have been found to inhibit a number of competitor species (reviewed in Patil et al., 2000) and play an important role in antagonistic fungal interactions. Recently, a chitinase from the biocontrol fungus *T. asperellum* PQ34 was observed to have a strong inhibitory effect on the growth of fungal pathogens on their plant hosts (*Sclerotium rolfsii* on peanut and *Colletotrichum* species on mango or chilli; Loc et al., 2020). Similar findings have also been made in *Trichoderma* sp. SANA20 (Aoki et al., 2020). Furthermore, deletion and/or disruption of genes encoding GH18 chitinases from the mycoparasitic biocontrol fungus *Clonostachys rosea* (*CrChiC2*: Tzelepis et al., 2015; *CrEch37*, *CrEch42*, and *CrEch58*: Mamarabadi et al., 2008) were found to reduce the inhibition of *B. cinerea* and *Fusarium culmorum*, respectively, in culture. While no such inhibition was observed *in planta*, this could be explained by the high degree of functional redundancy within GH18 chitinases (Langner and Göhre, 2016). However, further research is required to determine whether this is the case.

### Secreted Glycoside Hydrolase Family 25 (GH25) Proteins

Similar to the GH18 proteins described above, other secreted GH proteins of plant-associated fungi and oomycetes function as effectors with roles in manipulating plant microbiota during host colonization (Rovenich et al., 2014; Snelders et al., 2018). An example is MbA_GH25, a GH family 25 (GH25) protein with lysozyme activity of the epiphytic, basidiomycete yeast *Moesziomyces bullatus* ex *Albugo* (*MbA*; Eitzen et al., 2021). While *M. bullatus* is a smut pathogen of millet, *MbA* has been identified in the microbial phyllosphere of *A. thaliana*, where it showed antagonistic interactions with several bacteria (Eitzen et al., 2021). *MbA* was originally co-isolated with the oomycete white rust pathogen *Albugo laibachii*, which is the primary hub microbe of the *A. thaliana* phyllosphere (Agler et al., 2016). Interestingly, MbA strongly inhibited virulence of *A. laibachii* when co-inoculated onto *A. thaliana* leaves. RNA-sequencing of *MbA* identified the transcriptional induction of several GH-encoding genes upon contact with *A. laibachii* on the leaf surface (Eitzen et al., 2021). Strikingly, deletion of an *Albugo*-induced *GH25* gene, *MbA-GH25*, largely abolished the antagonistic activity of *MbA* towards *A. laibachii* (Eitzen et al., 2021). Similarly, enzymatically active, recombinant MbA-GH25 protein could significantly block *A. laibachii* infection of *A. thaliana*, demonstrating the biological function of this fungal lysozyme in microbial antagonism (Eitzen et al., 2021). Beyond this recent example, the biological functions of GH25 enzymes are poorly understood. Some of these hydrolases had been reported to be associated with microbial hyperparasitism in both fungal and oomycete species (Horner et al., 2012; Hyde et al., 2019). Since GH25 hydrolases can be found in many plant-associated fungi, future research will be necessary to elucidate the functions of these enzymes in the microbial leaf phyllosphere.

### Secreted Glycoside Hydrolase Family 28 (GH28) Proteins

Secreted GH family 28 (GH28) endopolygalacturonases (PGs; Supplementary Table 1), which hydrolyze the homogalacturonan domain of pectic polysaccharides present in plant cell walls, can also be recognized as MAMPs by plant PRRs. To date, PGs have been most extensively studied in *B. cinerea*, which carries six PG-encoding genes (*BcPG1–6*; Wubben et al., 1999). The expression of these genes *in planta* is dependent on both the infection stage and the host that is being colonized (ten Have et al., 2001). Early research highlighted a role for the PGs of *B. cinerea* in promoting host colonization, with mutants deleted for either the *BcPG1* or *BcPG2* gene displaying a strong reduction in virulence on tomato and broad bean (*Vicia faba*) leaves (ten Have et al., 1998; Kars et al., 2005). Subsequent research revealed that four PGs from *B. cinerea* (BcPG2, BcPG3, BcPG4, and BcPG6), as well as one PG from the fungal saprotroph *Aspergillus niger* (AnPGB), trigger cell death upon infiltration into leaves of *A. thaliana* accession Colombia (Zhang et al., 2014). This recognition was mediated by RLP42/RBPG1, an LRR-RLP PRR, with cell death elicitation dependent on SOBIR1 (Zhang et al., 2014). Consistent with these proteins being recognized as MAMPs, the cell death response was also triggered by a catalytically-inactivated form of BcPG3 (Zhang et al., 2014). Furthermore, RLP42 and BcPG3 were found to physically interact (Zhang et al., 2014). This recognition of *B. cinerea* PGs as MAMPs is not restricted to *A. thaliana*; BcPG1 is also able to induce defense responses such as ROS production in cell suspensions of grape (*Vitis vinifera*), independent of enzymatic activity (Poinssot et al., 2003).

More recently, it has been shown that a nine-amino acid fragment from BcPG6, pg9(At), which is conserved across fungal PGs and is a derivative of a slightly larger but equally active 13-amino acid fragment, pg13(At), is sufficient to activate RLP42-dependent immunity in *A. thaliana* (Zhang et al., 2021c). Indeed, in immune system activation experiments involving ethylene production, synthetic pg9(At) or pg13(At) peptides derived from BcPG6 (and BcPG2), as well as the other fungal PGs AnPGI, AnPGB and AnPGD from *A. niger*, CluPG1 from *Colletotrichum lupine* (hemibiotrophic fungal pathogen of lupin) and FmPGA from *Fusarium verticillioides* (hemibiotrophic fungal pathogen of maize), were active upon infiltration into leaves from *A. thaliana* accession Colombia (Zhang et al., 2021c). Pg9(At) derived from PGs of *Phytophthora* species, however, induced only residual ethylene production, suggesting that while PGs from this class of pathogens can be recognized, it is with lower efficiency (Zhang et al., 2021c). Consistent with the findings of Zhang et al. (2014), RLP42 bound the pg13(At) peptide, leading to the recruitment of SERK family members, including BAK1, for activation of plant immune responses (Zhang et al., 2021c). A structure–function analysis based on domain-swap experiments between recombinant proteins RLP42 and RLP40 (a paralog of RLP42 that is insensitive to PGs; Zhang et al., 2014), as well as domain deletion and amino acid substitution experiments, subsequently revealed that LRRs 3, 5, 7, and 10, as well as a region containing a 49-amino acid island domain, are required for pg9(At) recognition by RLP42 (Zhang et al., 2021c).

Interestingly, in an assessment of recognition across a range of plant species and accessions, only 16 of 52 *A. thaliana* accessions tested responded to pg13(At), while all 16 other plant species tested were unresponsive. This indicated that pg13(At) recognition is, so far, restricted to *A. thaliana*, albeit with notable within-species diversity (Zhang et al., 2021c). Strikingly, *Arabidopsis arenosa* and *Brassica rapa*, two Brassicaceae species closely related to *A. thaliana* that are unresponsive to pg13(At), but responsive to BcPG6, instead perceived the overlapping PG peptides pg20(Aa); (20-amino acid fragment) and pg36(Bra); (36-amino acid fragment), respectively (Zhang et al., 2021c). As these two peptides are structurally distinct from pg9(At), it was concluded that there are distinct recognition specificities for PGs within the Brassicaceae family (Zhang et al., 2021c).

In contrast to that described for the PGs of *B. cinerea* in *A. thaliana*, *A. arenosa* and *B. rapa*, the BcPG2 protein was found to trigger necrosis when infiltrated into broad bean leaves or transiently expressed in *N. benthamiana*. However, this activity could be abolished upon mutation of the enzymatic active site (Kars et al., 2005; Joubert et al., 2007). This suggested that, in some host species, the PGs of *B. cinerea* may release oligogalacturonides from plant cell wall pectin that are subsequently recognized as DAMPs by plant PRRs to activate the plant immune system.

It should be noted that plants produce extracellular LRR-containing polygalacturonase-inhibiting proteins (PGIPs), which specifically inhibit pathogen-secreted PGs (Liu et al., 2017). Examples include PvPGIP2 from bean, which inhibits BcPG1 from *B. cinerea*, and is associated with reduced colonization by this fungus in transgenic *A. thaliana* and *N. tabacum* plants overexpressing PvPGIP2 (Manfredini et al., 2005), as well as GhPGIP1 from cotton (*Gossypium hirsutum*), which interacts with VdPG1 and FovPG1 (albeit weakly) from *V. dahliae* and *F. oxysporum* f. sp. *vasinfectum* respectively, and provides enhanced susceptibility to these pathogens in cotton when transcriptionally silenced (Liu et al., 2017). Along these lines, silencing of *AcPGIP* from kiwifruit (*Actinidia chinensis*) has also recently been shown to result in increased susceptibility to *B. cinerea* (Li et al., 2021).

GH28 proteins have also been found to play important roles in the establishment of symbiotic interactions. One such example is a GH28-encoding gene from the ectomycorrhizal fungus *Laccaria bicolor*, *LbGH28A*, which was found to be induced during the formation of ectomycorrhiza in *Populus trichocarpa* (Veneault-Fourrey et al., 2014) and *P. tremula* x *alba* (Zhang et al., 2021b). Immunocytolocalisation of LbGH28A demonstrated that the protein was present at hyphal tips within the Hartig net (Zhang et al., 2021b), which is formed by a network of hyphae growing between the rhizodermal cells of the plant to create a symbiotic interface through which nutrients are exchanged (Becquer et al., 2019). Remarkably, *LbGH28* knockdown mutants of *L. bicolor* were found to be deficient in their ability to form Haritg nets (Zhang et al., 2021b). As LbGH28A was shown to be an active endopolygalacturonase with pectinase activity, Zhang et al. (2021b) suggest that LbGH28 may be involved in remodeling the middle lamella through pectin hydrolysis, thus playing an essential role in plant-fungal symbiosis.

### Secreted Glycoside Hydrolase Family 45 (GH45) Proteins

Outside of the GH11, GH12 and GH28 proteins, other GH family members are also recognized as MAMPs by plants. An example is EG1, a GH family 45 (GH45) endoglucanohydrolase from *Rhizoctonia solani*, a broad host-range, soil-borne, necrotrophic fungal pathogen (Ma et al., 2015a). The *EG1* gene is most highly expressed during the early stages of infection on maize (*Zea mays*), peaking at 2–3 days post-inoculation (dpi), with expression also detected at 4–7 dpi (Ma et al., 2015a). This expression coincided with cell death induction during infection of maize by *R. solani* (Ma et al., 2015a). Protein infiltration experiments showed that EG1, as well as a catalytically inactive form of this protein, can trigger cell death in leaves of maize, *N. tabacum* cv. NC89 and *A. thaliana*, indicating that EG1 is recognized as a MAMP, with defense-related genes shown to be upregulated in both maize and *N. tabacum* cv. NC89 (Ma et al., 2015a). Other defense responses, such as ROS accumulation and ethylene biosynthesis, were also observed when these proteins were applied to suspension-cultured cells of *N. tabacum* cv. NC89 (Ma et al., 2015a). It has since been shown that three amino acid residues in a seven-amino acid sequence within EG1 (S**PW**AVN**D**), as well as two amino acid residues in a five-amino acid sequence (G**C**S**R**K), are required for cell death induction in *N. benthamiana* (Guo et al., 2021). Structural modelling suggests that these regions of EG1 are surface-exposed, but structurally independent (Guo et al., 2021).

## Conclusions and Future Perspectives

A large body of research over many years has focused on understanding the role of small, mostly non-enzymatic, secreted proteins from plant-associated fungi and oomycetes in plant–microbe interactions. However, it is clear from this review that a role for secreted GH proteins in these interactions cannot be overlooked. Indeed, like a lot of the small secreted proteins described to date from plant-associated fungi and oomycetes, many secreted GH proteins from these microbial organisms also function as effectors to promote host colonization. Likewise, GH proteins can also act as (or produce) invasion patterns that activate the plant immune system to hinder host infection. Consequently, the identification and functional characterization of secreted GH proteins will be pivotal to our future understanding of how plant-associated fungi and oomycetes interact with their hosts at the molecular level to cause disease or trigger host resistance. Such an understanding is important, as it may inform disease control strategies. This could be mediated through, for example, the use of *PRR* genes active against secreted GH proteins. In the case of the PRR RXEG1, for instance, which recognizes GH12 proteins from various fungal and oomycete species, a contribution to plant immunity against the broad host-range oomycete pathogen *Phytophthora parasitica* was observed in *N. benthamiana* (Wang et al., 2018), suggesting it may be an interesting candidate for transfer to other plant species.

Much is still left to be learnt, however, about the full diversity of virulence functions performed by secreted GH proteins. In particular, it is not yet clear to what extent these proteins are involved in the remodeling of surface-associated carbohydrates present in fungal or oomycete cell walls, for example to enable modification of hyphal surfaces or infection structures required during plant colonization. Fujikawa et al. (2012), for instance, identified MoAGS1, a transmembrane α-1,3-glucan synthase from *M. oryzae* that carries an extracellular GH family 13 (GH13) domain. While MoAGS1 is essential for the pathogenicity of *M. oryzae* on rice, and is responsible for the accumulation of α-1,3-glucan on the cell wall surface of infection structures (likely to prevent the hydrolysis of fungal chitin and β-1,3-glucan by plant-derived hydrolytic enzymes, as well as the detection of these carbohydrates by the plant immune system; Fujikawa et al., 2012), the precise role of the GH13 domain in this protein remains uncertain. In any case, a better understanding of substrate specificity will provide further information on the virulence functions that secreted GH proteins from plant-associated fungi and oomycetes play in plant–microbe interactions.

More research is also required to better understand to what extent secreted GH proteins of plant-associated fungi and oomycetes interact synergistically with each other or other CAZymes to perform their roles, as has been shown for FgXyr1 (GH10) and FgPg1 (GH28) of *F. graminearum*, which function synergistically to promote virulence in soybean and wheat (Paccanaro et al., 2017). Indeed, several other CAZymes have now been shown to be important virulence factors of plant-associated fungi and oomycetes, including CEs (Gui et al., 2018) chitin deacetylases (Cord-Landwehr et al., 2016; Gao et al., 2019; Noorifar et al., 2021; Rizzi et al., 2021), PLs (Fu et al., 2015; Yang et al., 2018), and lytic polysaccharide monooxygenases (LPMOs; Sabbadin et al., 2021). Another class of proteins not yet classified as CAZymes, but that have a DUF3129 domain (called effectors with chitinase activity; EWCA), have also been recently implicated in fungal virulence (Martínez-Cruz et al., 2021). As an example of synergism outside of the GHs, a CE family 5 (CE5) cutinase from *V. dahliae*, VdCUT11, was found to trigger cell death and other defense responses in *N. benthamiana* only in the absence of VdCBM1, a carbohydrate-binding module family 1 (CBM1) protein from this fungus (Gui et al., 2017). It has been suggested that the defense responses triggered by VdCUT11 are the result of DAMP recognition, following the degradation of suberin in the roots of *N. benthamiana* by this enzyme, and that VdCBM1 suppresses these defense responses to promote host colonization (Gui et al., 2017).

The synergistic interaction between VdCUT11 and VdCBM1 raises an important issue. Many of the secreted GH proteins from plant-associated fungi and oomycetes that have been shown to trigger cell death or other defense responses in plants have been studied in isolation of the microorganisms from which they are derived, for example by using *Agrobacterium tumefaciens*-mediated transient expression assays (ATTAs) or protein infiltration experiments. As a consequence, these responses have been studied in the absence of other effectors that make up the microorganism’s full effector repertoire. This is important, because under natural infection conditions, other effectors in the repertoire may function to suppress or prevent the plant defense responses elicited by the secreted GH protein. An excellent example of this is the RXLR effector repertoire of *P. sojae*, from which 23 RXLR effectors were found to suppress XEG1-mediated cell death in *N. benthamiana* upon co-expression with XEG1 in ATTA experiments, including several known to be expressed within 30 min of host infection by *P. sojae* (Ma et al., 2015b).

In cases where *A. tumefaciens*-mediated transient expression assays or protein infiltration experiments have been used exclusively to determine whether a secreted GH triggers plant defense responses, care also needs to be taken as to whether the observed responses are biologically relevant or an artefact of over-production or excessive protein concentration. Under natural infection conditions, the secreted GH protein may never be produced in sufficient quantities to elicit plant defense responses. Moreover, responses that are observed in non-host plants might not be representative of responses observed in host plants; for example, secreted GH proteins may release a DAMP in the non-host plant that is not present in the host plant. Thus, ideally, secreted GH proteins should be functionally characterized in the context of the microorganism from which they are derived, and the natural host plant.

Another area of research that requires more attention involves understanding why some secreted GH proteins from the same GH family trigger strong plant defense responses (i.e., cell death), while others trigger only weak defense responses (e.g., ethylene production or ROS accumulation) or no response at all. This has been shown for several GH12 proteins of fungal and oomycete species (Ma et al., 2015b), as well as GH17 proteins from *C. fulvum* (Ökmen et al., 2019). In such cases, differences in the amino acid sequence of the family members might influence their tertiary structure, surface charge, glycosylation status, or stability in the plant environment, for example; these differences could translate into variations in substrate specificity (e.g., affecting DAMP release) or the affinity for cognate immune receptors and/or other host targets involved in plant defense. Certainly, using experiments based on ATTAs or protein infiltration, secreted GH proteins that only trigger weak plant defense responses have largely been overlooked to date, with preference tending to be given to those that instead trigger cell death. Experiments that focus on the identification of secreted GH proteins that do not trigger cell death, but instead induce responses such as ethylene production, ROS accumulation or the expression of defense-related genes, would provide a starting point for addressing this knowledge gap.

Finally, more research is required to better understand how secreted GH proteins, such as the BcCrh1 GH16 protein from *B. cinerea* (Bi et al., 2021), enter plant cells. Here, one line of enquiry could involve the delivery of these proteins by extracellular vesicles (EVs), given that fungal EV cargo often contains CAZymes, such as GHs with a signal peptide (e.g., Garcia-Ceron et al., 2021).

In recent years, huge leaps have been made in our understanding of the roles that secreted GH proteins from plant-associated fungi and oomycetes play in promoting host colonization or in activating the plant immune system. However, much is still to be learnt about the full diversity of roles played by this intriguing class of proteins, as well as the molecular mechanisms that underpin them. With more and more secreted GH proteins being identified, facilitated through the ever-increasing number and availability of new fungal and oomycete genomes, we anticipate that GH proteins will in future gain the same level of recognition as small secreted non-enzymatic proteins as critical effectors in plant-microbe interactions.

## Author Contributions

All authors listed have made a substantial, direct, and intellectual contribution to the work and approved it for publication.

## Funding

EB, RB, and CM are supported by the Tertiary Education Commission through the Centres of Research Excellence Program http://www.tec.govt.nz/funding/funding-and-performance/funding/fund-finder/centres-of-research-excellence/current-cores/.

## Conflict of Interest

The authors declare that the research was conducted in the absence of any commercial or financial relationships that could be construed as a potential conflict of interest.

## Publisher’s Note

All claims expressed in this article are solely those of the authors and do not necessarily represent those of their affiliated organizations, or those of the publisher, the editors and the reviewers. Any product that may be evaluated in this article, or claim that may be made by its manufacturer, is not guaranteed or endorsed by the publisher.

## Footnotes

^1^ http://www.cazy.org/

## References

[ref1] Agler M. T. Ruhe J. Kroll S. Morhenn C. Kim S.-T. Weigel D.. (2016). Microbial hub taxa link host and abiotic factors to plant microbiome variation. PLoS Biol. 14:e1002352. doi: 10.1371/journal.pbio.1002352, PMID: 26788878 PMC4720289

[ref2] Aoki Y. Haga S. Suzuki S. (2020). Direct antagonistic activity of chitinase produced by *Trichoderma* sp. SANA20 as biological control agent for grey mould caused by *Botrytis cinerea*. Cogent Biol. 6:1747903. doi: 10.1080/23312025.2020.1747903

[ref3] Avni A. Bailey B. A. Mattoo A. K. Anderson J. D. (1994). Induction of ethylene biosynthesis in *Nicotiana tabacum* by a *Trichoderma viride* xylanase is correlated to the accumulation of 1-aminocyclopropane-1-carboxylic acid (ACC) synthase and ACC oxidase transcripts. Plant Physiol. 106, 1049–1055. doi: 10.1104/pp.106.3.1049, PMID: 7824643 PMC159630

[ref4] Aziz A. Gauthier A. Bézier A. Poinssot B. Joubert J.-M. Pugin A.. (2007). Elicitor and resistance-inducing activities of β-1,4 cellodextrins in grapevine, comparison with β-1,3 glucans and α-1,4 oligogalacturonides. J. Exp. Bot. 58, 1463–1472. doi: 10.1093/jxb/erm008, PMID: 17322548

[ref5] Bailey B. A. Dean J. F. Anderson J. D. (1990). An ethylene biosynthesis-inducing endoxylanase elicits electrolyte leakage and necrosis in *Nicotiana tabacum* cv Xanthi leaves. Plant Physiol. 94, 1849–1854. doi: 10.1104/pp.94.4.1849, PMID: 16667926 PMC1077463

[ref6] Bailey B. A. Korcak R. F. Anderson J. D. (1992). Alterations in *Nicotiana tabacum* L. cv Xanthi cell membrane function following treatment with an ethylene biosynthesis-inducing endoxylanase. Plant Physiol. 100, 749–755. doi: 10.1104/pp.100.2.749, PMID: 16653055 PMC1075622

[ref7] Bailey B. A. Korcak R. F. Anderson J. D. (1993). Sensitivity to an ethylene biosynthesis-inducing endoxylanase in *Nicotiana tabacum* L. cv Xanthi is controlled by a single dominant gene. Plant Physiol. 101, 1081–1088. doi: 10.1104/pp.101.3.1081, PMID: 12231760 PMC158728

[ref8] Bangham A. D. Horne R. W. (1962). Action of saponin on biological cell membranes. Nature 196, 952–953. doi: 10.1038/196952a0 13966357

[ref9] Bar M. Avni A. (2009). EHD2 inhibits ligand-induced endocytosis and signaling of the leucine-rich repeat receptor-like protein LeEix2. Plant J. 59, 600–611. doi: 10.1111/j.1365-313X.2009.03897.x, PMID: 19392695

[ref10] Bar M. Sharfman M. Avni A. (2011). LeEix1 functions as a decoy receptor to attenuate LeEix2 signaling. Plant Signal. Behav. 6, 455–457. doi: 10.4161/psb.6.3.14714, PMID: 21364318 PMC3142438

[ref11] Bar M. Sharfman M. Ron M. Avni A. (2010). BAK1 is required for the attenuation of ethylene-inducing xylanase (Eix)-induced defense responses by the decoy receptor LeEix1. Plant J. 63, 791–800. doi: 10.1111/j.1365-313X.2010.04282.x, PMID: 20561260

[ref12] Becker M. Becker Y. Green K. Scott B. (2016). The endophytic symbiont *Epichloë festucae* establishes an epiphyllous net on the surface of *Lolium perenne* leaves by development of an expressorium, an appressorium-like leaf exit structure. New Phytol. 211, 240–254. doi: 10.1111/nph.13931, PMID: 26991322 PMC5069595

[ref13] Becquer A. Guerrero-Galán C. Eibensteiner J. L. Houdinet G. Bücking H. Zimmermann S. D.. (2019). “Chapter 3: The ectomycorrhizal contribution to tree nutrition,” in Advances in Botanical Research. Vol. 89. ed. Cánovas F. M. (Cambridge, MA, USA: Academic Press), 77–126.

[ref14] Bellincampi D. Cervone F. Lionetti V. (2014). Plant cell wall dynamics and wall-related susceptibility in plant–pathogen interactions. Front. Plant Sci. 5:228. doi: 10.3389/fpls.2014.00228, PMID: 24904623 PMC4036129

[ref15] Benedetti M. Pontiggia D. Raggi S. Cheng Z. Scaloni F. Ferrari S.. (2015). Plant immunity triggered by engineered in vivo release of oligogalacturonides, damage-associated molecular patterns. Proc. Natl. Acad. Sci. 112, 5533–5538. doi: 10.1073/pnas.1504154112, PMID: 25870275 PMC4418913

[ref16] Bi K. Scalschi L. Jaiswal N. Mengiste T. Fried R. Sanz A. B.. (2021). The *Botrytis cinerea* Crh1 transglycosylase is a cytoplasmic effector triggering plant cell death and defense response. Nat. Commun. 12:2166. doi: 10.1038/s41467-021-22436-1, PMID: 33846308 PMC8042016

[ref17] Bouarab K. Melton R. Peart J. Baulcombe D. Osbourn A. (2002). A saponin-detoxifying enzyme mediates suppression of plant defences. Nature 418, 889–892. doi: 10.1038/nature00950, PMID: 12192413

[ref18] Bowyer P. Clarke B. R. Lunness P. Daniels M. J. Osbourn A. E. (1995). Host range of a plant pathogenic fungus determined by a saponin detoxifying enzyme. Science 267, 371–374. doi: 10.1126/science.7824933, PMID: 7824933

[ref19] Brito N. Espino J. J. González C. (2006). The endo-β-1, 4-xylanase Xyn11A is required for virulence in *Botrytis cinerea*. Mol. Plant-Microbe Interact. 19, 25–32. doi: 10.1094/MPMI-19-0025, PMID: 16404950

[ref20] Brutus A. Reca I. B. Herga S. Mattei B. Puigserver A. Chaix J. C.. (2005). A family 11 xylanase from the pathogen *Botrytis cinerea* is inhibited by plant endoxylanase inhibitors XIP-I and TAXI-I. Biochem. Biophys. Res. Commun. 337, 160–166. doi: 10.1016/j.bbrc.2005.09.030, PMID: 16185656

[ref21] Brutus A. Sicilia F. Macone A. Cervone F. De Lorenzo G. (2010). A domain swap approach reveals a role of the plant wall-associated kinase 1 (WAK1) as a receptor of oligogalacturonides. Proc. Natl. Acad. Sci. 107, 9452–9457. doi: 10.1073/pnas.1000675107, PMID: 20439716 PMC2889104

[ref22] Cao Y. Liang Y. Tanaka K. Nguyen C. T. Jedrzejczak R. P. Joachimiak A.. (2014). The kinase LYK5 is a major chitin receptor in *Arabidopsis* and forms a chitin-induced complex with related kinase CERK1. elife 3:e03766. doi: 10.7554/eLife.03766, PMID: 25340959 PMC4356144

[ref23] Carrión V. J. Perez-Jaramillo J. Cordovez V. Tracanna V. de Hollander M. Ruiz-Buck D.. (2019). Pathogen-induced activation of disease-suppressive functions in the endophytic root microbiome. Science 366, 606–612. doi: 10.1126/science.aaw9285, PMID: 31672892

[ref24] Claverie J. Balacey S. Lemaître-Guillier C. Brulé D. Chiltz A. Granet L.. (2018). The cell wall-derived xyloglucan is a new DAMP triggering plant immunity in *Vitis vinifera* and *Arabidopsis thaliana*. Front. Plant Sci. 9:1725. doi: 10.3389/fpls.2018.01725, PMID: 30546374 PMC6280107

[ref25] Cook D. E. Mesarich C. H. Thomma B. P. (2015). Understanding plant immunity as a surveillance system to detect invasion. Annu. Rev. Phytopathol. 53, 541–563. doi: 10.1146/annurev-phyto-080614-120114, PMID: 26047564

[ref26] Cord-Landwehr S. Melcher R. L. Kolkenbrock S. Moerschbacher B. M. (2016). A chitin deacetylase from the endophytic fungus *Pestalotiopsis* sp. efficiently inactivates the elicitor activity of chitin oligomers in rice cells. Sci. Rep. 6, 1–11. doi: 10.1038/srep38018, PMID: 27901067 PMC5128826

[ref27] Couturier M. Navarro D. Olivé C. Chevret D. Haon M. Favel A.. (2012). Post-genomic analyses of fungal lignocellulosic biomass degradation reveal the unexpected potential of the plant pathogen *Ustilago maydis*. BMC Genomics 13:57. doi: 10.1186/1471-2164-13-57, PMID: 22300648 PMC3298532

[ref28] Crombie W. M. L. Crombie L. Green J. B. Lucas J. A. (1986). Pathogenicity of ‘take-all’ fungus to oats: its relationship to the concentration and detoxification of the four avenacins. Phytochemistry 25, 2075–2083. doi: 10.1016/0031-9422(86)80069-3

[ref29] de Azevedo Souza C. A. Li S. Lin A. Z. Boutrot F. Grossmann G. Zipfel C.. (2017). Cellulose-derived oligomers act as damage-associated molecular patterns and trigger defense-like responses. Plant Physiol. 173, 2383–2398. doi: 10.1104/pp.16.01680, PMID: 28242654 PMC5373054

[ref30] Dean J. F. Anderson J. D. (1991). Ethylene biosynthesis-inducing xylanase: II. Purification and physical characterization of the enzyme produced by *Trichoderma viride*. Plant Physiol. 95, 316–323. doi: 10.1104/pp.95.1.316, PMID: 16667971 PMC1077524

[ref31] Dean J. Gamble H. Anderson J. (1989). The ethylene biosynthesis-inducing xylanase: its induction in *Trichoderma viride* and certain plant pathogens. Phytopathology 79, 1071–1078. doi: 10.1094/Phyto-79-1071

[ref32] Dickman M. B. Fluhr R. (2013). Centrality of host cell death in plant-microbe interactions. Annu. Rev. Phytopathol. 51, 543–570. doi: 10.1146/annurev-phyto-081211-173027, PMID: 23915134

[ref33] Doehlemann G. Hemetsberger C. (2013). Apoplastic immunity and its suppression by filamentous plant pathogens. New Phytol. 198, 1001–1016. doi: 10.1111/nph.12277, PMID: 23594392

[ref34] Drula E. Garron M. L. Dogan S. Lombard V. Henrissat B. Terrapon N. (2022). The carbohydrate-active enzyme database: functions and literature. Nucleic Acids Res. 50, D571–d577. doi: 10.1093/nar/gkab1045, PMID: 34850161 PMC8728194

[ref35] Eitzen K. Sengupta P. Kroll S. Kemen E. Doehlemann G. (2021). A fungal member of the *Arabidopsis thaliana* phyllosphere antagonizes *Albugo laibachii* via a GH25 lysozyme. elife 10:e65306. doi: 10.7554/eLife.65306, PMID: 33427195 PMC7870139

[ref36] El Gueddari N. E. Rauchhaus U. Moerschbacher B. M. Deising H. B. (2002). Developmentally regulated conversion of surface-exposed chitin to chitosan in cell walls of plant pathogenic fungi. New Phytol. 156, 103–112. doi: 10.1046/j.1469-8137.2002.00487.x

[ref37] Ferrari S. Savatin D. Sicilia F. Gramegna G. Cervone F. De Lorenzo G. (2013). Oligogalacturonides: plant damage-associated molecular patterns and regulators of growth and development. Front. Plant Sci. 4:49. doi: 10.3389/fpls.2013.00049, PMID: 23493833 PMC3595604

[ref38] Fesel P. H. Zuccaro A. (2016). β-Glucan: crucial component of the fungal cell wall and elusive MAMP in plants. Fungal Genet. Biol. 90, 53–60. doi: 10.1016/j.fgb.2015.12.004, PMID: 26688467

[ref39] Fiorin G. L. Sanchéz-Vallet A. Thomazella D. P. d. T. do Prado P. F. V. do Nascimento L. C. Figueira A. V. d. O.. (2018). Suppression of plant immunity by fungal chitinase-like effectors. Curr. Biol. 28, 3023–3030.e5. doi: 10.1016/j.cub.2018.07.055, PMID: 30220500

[ref40] Fluhr R. Sessa G. Sharon A. Ori N. Lotan T. (1991). “Pathogenesis-related proteins exhibit both pathogen-induced and developmental regulation,” in Advances in Molecular Genetics of Plant-Microbe Interactions. Vol. 1. eds. H. Hennecke and D. P. S. Verma (Dordrecht: Springer), 387–394.

[ref41] Frías M. González M. González C. Brito N. (2019). A 25-residue peptide from *Botrytis cinerea* xylanase BcXyn11A elicits plant defenses. Front. Plant Sci. 10:474. doi: 10.3389/fpls.2019.00474, PMID: 31057580 PMC6477079

[ref42] Fu L. Zhu C. Ding X. Yang X. Morris P. F. Tyler B. M.. (2015). Characterization of cell-death-inducing members of the pectate lyase gene family in *Phytophthora capsici* andt their contributions to infection of pepper. Mol. Plant-Microbe Interact. 28, 766–775. doi: 10.1094/mpmi-11-14-0352-r, PMID: 25775270

[ref43] Fuchs Y. Anderson J. D. (1987). Purification and characterization of ethylene inducing proteins from cellulysin. Plant Physiol. 84, 732–736. doi: 10.1104/pp.84.3.732, PMID: 16665512 PMC1056660

[ref44] Fuchs Y. Saxena A. Gamble H. R. Anderson J. D. (1989). Ethylene biosynthesis-inducing protein from cellulysin is an endoxylanase 1. Plant Physiol. 89, 138–143. doi: 10.1104/pp.89.1.138, PMID: 16666504 PMC1055809

[ref45] Fujikawa T. Kuga Y. Yano S. Yoshimi A. Tachiki T. Abe K.. (2009). Dynamics of cell wall components of *Magnaporthe grisea* during infectious structure development. Mol. Microbiol. 73, 553–570. doi: 10.1111/j.1365-2958.2009.06786.x, PMID: 19602150

[ref46] Fujikawa T. Sakaguchi A. Nishizawa Y. Kouzai Y. Minami E. Yano S.. (2012). Surface α-1,3-glucan facilitates fungal stealth infection by interfering with innate immunity in plants. PLoS Pathog. 8:e1002882. doi: 10.1371/journal.ppat.1002882, PMID: 22927818 PMC3426526

[ref47] Furman-Matarasso N. Cohen E. Du Q. Chejanovsky N. Hanania U. Avni A. (1999). A point mutation in the ethylene-inducing xylanase elicitor inhibits the β-1-4-endoxylanase activity but not the elicitation activity. Plant Physiol. 121, 345–352. doi: 10.1104/pp.121.2.345, PMID: 10517825 PMC59396

[ref48] Gao F. Zhang B.-S. Zhao J.-H. Huang J.-F. Jia P.-S. Wang S.. (2019). Deacetylation of chitin oligomers increases virulence in soil-borne fungal pathogens. Nat. Plants 5, 1167–1176. doi: 10.1038/s41477-019-0527-4, PMID: 31636399

[ref49] Garcia-Ceron D. Lowe R. G. T. McKenna J. A. Brain L. M. Dawson C. S. Clark B.. (2021). Extracellular vesicles from *fusarium graminearum* contain protein effectors expressed during infection of corn. J. Fungi 7:977. doi: 10.3390/jof7110977, PMID: 34829264 PMC8625442

[ref50] Gow N. A. R. Latge J. P. Munro C. A. (2017). The fungal cell wall: structure, biosynthesis, and function. Microbiol. Spectr. 4. doi: 10.1128/microbiolspec.FUNK-0035-2016, PMID: 28513415 PMC11687499

[ref51] Gui Y. J. Chen J. Y. Zhang D. D. Li N. Y. Li T. G. Zhang W. Q.. (2017). *Verticillium dahliae* manipulates plant immunity by glycoside hydrolase 12 proteins in conjunction with carbohydrate-binding module 1. Environ. Microbiol. 19, 1914–1932. doi: 10.1111/1462-2920.13695, PMID: 28205292

[ref52] Gui Y.-J. Zhang W.-Q. Zhang D.-D. Zhou L. Short D. P. G. Wang J.. (2018). A *Verticillium dahliae* extracellular cutinase modulates plant immune responses. Mol. Plant-Microbe Interact. 31, 260–273. doi: 10.1094/mpmi-06-17-0136-r, PMID: 29068240

[ref53] Guo X. Liu N. Zhang Y. Chen J. (2021). Pathogen-associated molecular pattern active sites of GH45 endoglucanohydrolase from *Rhizoctonia solani*. Phytopathology. doi: 10.1094/PHYTO-04-21-0164-R, PMID: [Epub ahead of print] 34165320

[ref54] Han Y. Song L. Peng C. Liu X. Liu L. Zhang Y.. (2019). A *Magnaporthe* chitinase interacts with a rice jacalin-related lectin to promote host colonization. Plant Physiol. 179, 1416–1430. doi: 10.1104/pp.18.01594, PMID: 30696749 PMC6446787

[ref55] Hane J. K. Paxman J. Jones D. A. B. Oliver R. P. de Wit P. (2020). “CATAStrophy,” a genome-informed trophic classification of filamentous plant pathogens – how many different types of filamentous plant pathogens are there? Front. Microbiol. 10:3088. doi: 10.3389/fmicb.2019.03088, PMID: 32038539 PMC6986263

[ref56] He Q. McLellan H. Boevink P. C. Birch P. R. J. (2020). All roads lead to susceptibility: The many modes of action of fungal and oomycete intracellular effectors. Plant Commun. 1:100050. doi: 10.1016/j.xplc.2020.100050, PMID: 33367246 PMC7748000

[ref57] Henrissat B. (1991). A classification of glycosyl hydrolases based on amino acid sequence similarities. Biochem. J. 280, 309–316. doi: 10.1042/bj2800309, PMID: 1747104 PMC1130547

[ref58] Henrissat B. Davies G. (1997). Structural and sequence-based classification of glycoside hydrolases. Curr. Opin. Struct. Biol. 7, 637–644. doi: 10.1016/s0959-440x(97)80072-3, PMID: 9345621

[ref59] Horner N. R. Grenville-Briggs L. J. van West P. (2012). The oomycete *Pythium oligandrum* expresses putative effectors during mycoparasitism of *Phytophthora infestans* and is amenable to transformation. Fungal Biol. 116, 24–41. doi: 10.1016/j.funbio.2011.09.004, PMID: 22208599

[ref60] Hou S. Liu Z. Shen H. Wu D. (2019). Damage-associated molecular pattern-triggered immunity in plants. Front. Plant Sci. 10:646. doi: 10.3389/fpls.2019.00646, PMID: 31191574 PMC6547358

[ref61] Hyde K. D. Xu J. Rapior S. Jeewon R. Lumyong S. Niego A. G. T.. (2019). The amazing potential of fungi: 50 ways we can exploit fungi industrially. Fungal Divers. 97, 1–136. doi: 10.1007/s13225-019-00430-9

[ref62] Jacoby R. P. Koprivova A. Kopriva S. (2020). Pinpointing secondary metabolites that shape the composition and function of the plant microbiome. J. Exp. Bot. 72, 57–69. doi: 10.1093/jxb/eraa424, PMID: 32995888 PMC7816845

[ref63] Joubert D. A. Kars I. Wagemakers L. Bergmann C. Kemp G. Vivier M. A.. (2007). A polygalacturonase-inhibiting protein from grapevine reduces the symptoms of the endopolygalacturonase BcPG2 from *Botrytis cinerea* in *Nicotiana benthamiana* leaves without any evidence for in vitro interaction. Mol. Plant-Microbe Interact. 20, 392–402. doi: 10.1094/MPMI-20-4-0392, PMID: 17427809

[ref64] Kars I. Krooshof G. H. Wagemakers L. Joosten R. Benen J. A. E. Van Kan J. A. L. (2005). Necrotizing activity of five *Botrytis cinerea* endopolygalacturonases produced in *Pichia pastoris*. Plant J. 43, 213–225. doi: 10.1111/j.1365-313X.2005.02436.x, PMID: 15998308

[ref65] Kubicek C. P. Starr T. L. Glass N. L. (2014). Plant cell wall-degrading enzymes and their secretion in plant-pathogenic fungi. Annu. Rev. Phytopathol. 52, 427–451. doi: 10.1146/annurev-phyto-102313-045831, PMID: 25001456

[ref66] Kuć J. (1997). Molecular aspects of plant responses to pathogens. Acta Physiol. Plant. 19, 551–559. doi: 10.1007/s11738-997-0053-2

[ref67] Kumar M. Turner S. (2015). “Cell wall biosynthesis,” in eLS. (Chichester: John Wiley & Sons), 1–11.

[ref68] Langner T. Göhre V. (2016). Fungal chitinases: function, regulation, and potential roles in plant/pathogen interactions. Curr. Genet. 62, 243–254. doi: 10.1007/s00294-015-0530-x, PMID: 26527115

[ref69] Laxalt A. M. Raho N. Ten Have A. Lamattina L. (2007). Nitric oxide is critical for inducing phosphatidic acid accumulation in xylanase-elicited tomato cells. J. Biol. Chem. 282, 21160–21168. doi: 10.1074/jbc.M701212200, PMID: 17491015

[ref70] Li Z.-X. Chen M. Miao Y.-X. Li Q. Ren Y. Zhang W.-L.. (2021). The role of AcPGIP in the kiwifruit (*Actinidia chinensis*) response to *Botrytis cinerea*. Funct. Plant Biol. 48, 1254–1263. doi: 10.1071/FP21054, PMID: 34600600

[ref71] Li J. Wen J. Lease K. A. Doke J. T. Tax F. E. Walker J. C. (2002). BAK1, an *Arabidopsis* LRR receptor-like protein kinase, interacts with BRI1 and modulates brassinosteroid signaling. Cell 110, 213–222. doi: 10.1016/s0092-8674(02)00812-7, PMID: 12150929

[ref72] Liebrand T. W. H. van den Berg G. C. M. Zhang Z. Smit P. Cordewener J. H. G. America A. H. P.. (2013). Receptor-like kinase SOBIR1/EVR interacts with receptor-like proteins in plant immunity against fungal infection. Proc. Natl. Acad. Sci. 110, 10010–10015. doi: 10.1073/pnas.1220015110, PMID: 23716655 PMC3683720

[ref73] Liebrand T. W. H. van den Burg H. A. Joosten M. H. A. J. (2014). Two for all: receptor-associated kinases SOBIR1 and BAK1. Trends Plant Sci. 19, 123–132. doi: 10.1016/j.tplants.2013.10.003, PMID: 24238702

[ref74] Liu N. Zhang X. Sun Y. Wang P. Li X. Pei Y.. (2017). Molecular evidence for the involvement of a polygalacturonase-inhibiting protein, GhPGIP1, in enhanced resistance to *Verticillium* and *fusarium* wilts in cotton. Sci. Rep. 7:39840. doi: 10.1038/srep39840, PMID: 28079053 PMC5228132

[ref75] Loc N. H. Huy N. D. Quang H. T. Lan T. T. Thu Ha T. T. (2020). Characterisation and antifungal activity of extracellular chitinase from a biocontrol fungus, *Trichoderma asperellum* PQ34. Mycology 11, 38–48. doi: 10.1080/21501203.2019.1703839, PMID: 32128280 PMC7033689

[ref76] Lombard V. Golaconda Ramulu H. Drula E. Coutinho P. M. Henrissat B. (2014). The carbohydrate-active enzymes database (CAZy) in 2013. Nucleic Acids Res. 42, D490–D495. doi: 10.1093/nar/gkt1178, PMID: 24270786 PMC3965031

[ref77] Lotan T. Fluhr R. (1990). Xylanase, a novel elicitor of pathogenesis-related proteins in tobacco, uses a non-ethylene pathway for induction 1. Plant Physiol. 93, 811–817. doi: 10.1104/pp.93.2.811, PMID: 16667541 PMC1062588

[ref78] Ma Y. Han C. Chen J. Li H. He K. Liu A.. (2015a). Fungal cellulase is an elicitor but its enzymatic activity is not required for its elicitor activity. Mol. Plant Pathol. 16, 14–26. doi: 10.1111/mpp.12156, PMID: 24844544 PMC6638370

[ref79] Ma Z. C. Song T. Q. Zhu L. Ye W. W. Wang Y. Shao Y. Y.. (2015b). A *Phytophthora sojae* glycoside hydrolase 12 protein is a major virulence factor during soybean infection and is recognized as a PAMP. Plant Cell 27, 2057–2072. doi: 10.1105/tpc.15.00390, PMID: 26163574 PMC4531360

[ref80] Ma Z. C. Zhu L. Song T. Q. Wang Y. Zhang Q. Xia Y. Q.. (2017). A paralogous decoy protects *Phytophthora sojae* apoplastic effector PsXEG1 from a host inhibitor. Science 355, 710–714. doi: 10.1126/science.aai7919, PMID: 28082413

[ref81] Mamarabadi M. Jensen B. Lübeck M. (2008). Three endochitinase-encoding genes identified in the biocontrol fungus *Clonostachys rosea* are differentially expressed. Curr. Genet. 54, 57–70. doi: 10.1007/s00294-008-0199-5, PMID: 18574585

[ref82] Manfredini C. Sicilia F. Ferrari S. Pontiggia D. Salvi G. Caprari C.. (2005). Polygalacturonase-inhibiting protein 2 of *Phaseolus vulgaris* inhibits BcPG1, a polygalacturonase of *Botrytis cinerea* important for pathogenicity, and protects transgenic plants from infection. Physiol. Mol. Plant Pathol. 67, 108–115. doi: 10.1016/j.pmpp.2005.10.002

[ref83] Martin F. Kohler A. Murat C. Balestrini R. Coutinho P. M. Jaillon O.. (2010). Périgord black truffle genome uncovers evolutionary origins and mechanisms of symbiosis. Nature 464, 1033–1038. doi: 10.1038/nature08867, PMID: 20348908

[ref84] Martínez-Cruz J. Romero D. Hierrezuelo J. Thon M. de Vicente A. Pérez-García A. (2021). Effectors with chitinase activity (EWCAs), a family of conserved, secreted fungal chitinases that suppress chitin-triggered immunity. Plant Cell 33, 1319–1340. doi: 10.1093/plcell/koab011, PMID: 33793825

[ref85] Martin-Hernandez A. M. Dufresne M. Hugouvieux V. Melton R. Osbourn A. (2000). Effects of targeted replacement of the tomatinase gene on the interaction of *Septoria lycopersici* with tomato plants. Mol. Plant-Microbe Interact. 13, 1301–1311. doi: 10.1094/mpmi.2000.13.12.1301, PMID: 11106022

[ref86] Mélida H. Bacete L. Ruprecht C. Rebaque D. del Hierro I. López G.. (2020). Arabinoxylan-oligosaccharides act as damage associated molecular patterns in plants regulating disease resistance. Front. Plant Sci. 11:1210. doi: 10.3389/fpls.2020.01210, PMID: 32849751 PMC7427311

[ref87] Mélida H. Sopeña-Torres S. Bacete L. Garrido-Arandia M. Jordá L. López G.. (2018). Non-branched β-1,3-glucan oligosaccharides trigger immune responses in *Arabidopsis*. Plant J. 93, 34–49. doi: 10.1111/tpj.13755, PMID: 29083116

[ref88] Miya A. Albert P. Shinya T. Desaki Y. Ichimura K. Shirasu K.. (2007). CERK1, a LysM receptor kinase, is essential for chitin elicitor signaling in *Arabidopsis*. Proc. Natl. Acad. Sci. 104, 19613–19618. doi: 10.1073/pnas.0705147104, PMID: 18042724 PMC2148337

[ref89] Newman M.-A. Sundelin T. Nielsen J. Erbs G. (2013). MAMP (microbe-associated molecular pattern)-triggered immunity in plants. Front. Plant Sci. 4:139. doi: 10.3389/fpls.2013.00139, PMID: 23720666 PMC3655273

[ref90] Noda J. Brito N. González C. (2010). The *Botrytis cinerea* xylanase Xyn11A contributes to virulence with its necrotizing activity, not with its catalytic activity. BMC Plant Biol. 10:38. doi: 10.1186/1471-2229-10-38, PMID: 20184750 PMC2844071

[ref91] Noorifar N. Savoian M. S. Ram A. Lukito Y. Hassing B. Weikert T. W.. (2021). Chitin deacetylases are required for *Epichloë festucae* endophytic cell wall remodeling during establishment of a mutualistic symbiotic interaction with *Lolium perenne*. Mol. Plant-Microbe Interact. 34, 1181–1192. doi: 10.1094/mpmi-12-20-0347-r, PMID: 34058838

[ref92] Ökmen B. Bachmann D. De Wit P. J. G. M. (2019). A conserved GH17 glycosyl hydrolase from plant pathogenic Dothideomycetes releases a DAMP causing cell death in tomato. Mol. Plant Pathol. 20, 1710–1721. doi: 10.1111/mpp.12872, PMID: 31603622 PMC6859711

[ref93] Ökmen B. Doehlemann G. (2014). Inside plant: biotrophic strategies to modulate host immunity and metabolism. Curr. Opin. Plant Biol. 20, 19–25. doi: 10.1016/j.pbi.2014.03.011, PMID: 24780462

[ref94] Ökmen B. Etalo D. W. Joosten M. H. A. J. Bouwmeester H. J. de Vos R. C. H. Collemare J.. (2013). Detoxification of α-tomatine by *Cladosporium fulvum* is required for full virulence on tomato. New Phytol. 198, 1203–1214. doi: 10.1111/nph.12208, PMID: 23448507

[ref95] Oliveira-Garcia E. Deising H. B. (2013). Infection structure-specific expression of β-1,3-glucan synthase is essential for pathogenicity of *Colletotrichum graminicola* and evasion of β-glucan-triggered immunity in maize. Plant Cell 25, 2356–2378. doi: 10.1105/tpc.112.103499, PMID: 23898035 PMC3723631

[ref96] Oliveira-Garcia E. Deising H. B. (2016). Attenuation of PAMP-triggered immunity in maize requires down-regulation of the key β-1,6-glucan synthesis genes *KRE5* and *KRE6* in biotrophic hyphae of *Colletotrichum graminicola*. Plant J. 87, 355–375. doi: 10.1111/tpj.13205, PMID: 27144995

[ref97] Osbourn A. (1996). Saponins and plant defence—a soap story. Trends Plant Sci. 1, 4–9. doi: 10.1016/S1360-1385(96)80016-1

[ref98] Paccanaro M. C. Sella L. Castiglioni C. Giacomello F. Martínez-Rocha A. L. D’Ovidio R.. (2017). Synergistic effect of different plant cell wall–degrading enzymes is important for virulence of *fusarium graminearum*. Mol. Plant-Microbe Interact. 30, 886–895. doi: 10.1094/MPMI-07-17-0179-R, PMID: 28800710

[ref99] Pareja-Jaime Y. Roncero M. I. G. Ruiz-Roldan M. C. (2008). Tomatinase from *fusarium oxysporum* f. sp *lycopersici* is required for full virulence on tomato plants. Mol. Plant-Microbe Interact. 21, 728–736. doi: 10.1094/Mpmi-21-6-0728, PMID: 18624637

[ref100] Patil R. S. Ghormade V. Deshpande M. V. (2000). Chitinolytic enzymes: an exploration. Enzym. Microb. Technol. 26, 473–483. doi: 10.1016/S0141-0229(00)00134-4, PMID: 10771049

[ref101] Poinssot B. Vandelle E. Bentéjac M. Adrian M. Levis C. Brygoo Y.. (2003). The endopolygalacturonase 1 from *Botrytis cinerea* activates grapevine defense reactions unrelated to its enzymatic activity. Mol. Plant-Microbe Interact. 16, 553–564. doi: 10.1094/mpmi.2003.16.6.553, PMID: 12795381

[ref102] Popper Z. A. Michel G. Hervé C. Domozych D. S. Willats W. G. T. Tuohy M. G.. (2011). Evolution and diversity of plant cell walls: From algae to flowering plants. Annu. Rev. Plant Biol. 62, 567–590. doi: 10.1146/annurev-arplant-042110-103809, PMID: 21351878

[ref103] Raaymakers T. M. Van den Ackerveken G. (2016). Extracellular recognition of oomycetes during biotrophic infection of plants. Front. Plant Sci. 7:906. doi: 10.3389/fpls.2016.00906, PMID: 27446136 PMC4915311

[ref104] Rafiei V. Vélëz H. Tzelepis G. (2021). The role of glycoside hydrolases in phytopathogenic fungi and oomycetes virulence. Int. J. Mol. Sci. 22:9359. doi: 10.3390/ijms22179359, PMID: 34502268 PMC8431085

[ref105] Rebaque D. Del Hierro I. López G. Bacete L. Vilaplana F. Dallabernardina P.. (2021). Cell wall-derived mixed-linked β-1,3/1,4-glucans trigger immune responses and disease resistance in plants. Plant J. 106, 601–615. doi: 10.1111/tpj.15185, PMID: 33544927 PMC8252745

[ref106] Rizzi Y. S. Happel P. Lenz S. Urs M. J. Bonin M. Cord-Landwehr S.. (2021). Chitosan and chitin deacetylase activity are necessary for development and virulence of *Ustilago maydis*. MBio 12, e03419–e03420. doi: 10.1128/mBio.03419-20 33653886 PMC8092297

[ref107] Rocafort M. Fudal I. Mesarich C. H. (2020). Apoplastic effector proteins of plant-associated fungi and oomycetes. Curr. Opin. Plant Biol. 56, 9–19. doi: 10.1016/j.pbi.2020.02.004, PMID: 32247857

[ref108] Ron M. Avni A. (2004). The receptor for the fungal elicitor ethylene-inducing xylanase is a member of a resistance-like gene family in tomato. Plant Cell 16, 1604–1615. doi: 10.1105/tpc.022475, PMID: 15155877 PMC490049

[ref109] Ron M. Kantety R. Martin G. Avidan N. Eshed Y. Zamir D.. (2000). High-resolution linkage analysis and physical characterization of the EIX-responding locus in tomato. Theor. Appl. Genet. 100, 184–189. doi: 10.1007/s001220050025

[ref110] Rotblat B. Enshell-Seijffers D. Gershoni J. M. Schuster S. Avni A. (2002). Identification of an essential component of the elicitation active site of the EIX protein elicitor. Plant J. 32, 1049–1055. doi: 10.1046/j.1365-313X.2002.01490.x, PMID: 12492845

[ref111] Rovenich H. Boshoven J. C. Thomma B. P. H. J. (2014). Filamentous pathogen effector functions: of pathogens, hosts and microbiomes. Curr. Opin. Plant Biol. 20, 96–103. doi: 10.1016/j.pbi.2014.05.001, PMID: 24879450

[ref112] Rovenich H. Zuccaro A. Thomma B. P. (2016). Convergent evolution of filamentous microbes towards evasion of glycan-triggered immunity. New Phytol. 212, 896–901. doi: 10.1111/nph.14064, PMID: 27329426

[ref113] Rui Y. Dinneny J. R. (2020). A wall with integrity: surveillance and maintenance of the plant cell wall under stress. New Phytol. 225, 1428–1439. doi: 10.1111/nph.16166 31486535

[ref114] Sabbadin F. Urresti S. Henrissat B. Avrova A. O. Welsh L. R. Lindley P. J.. (2021). Secreted pectin monooxygenases drive plant infection by pathogenic oomycetes. Science 373, 774–779. doi: 10.1126/science.abj1342, PMID: 34385392

[ref115] Sánchez-Vallet A. Mesters J. R. Thomma B. P. (2015). The battle for chitin recognition in plant-microbe interactions. FEMS Microbiol. Rev. 39, 171–183. doi: 10.1093/femsre/fuu003, PMID: 25725011

[ref116] Sattelmacher B. (2001). The apoplast and its significance for plant mineral nutrition. New Phytol. 149, 167–192. doi: 10.1046/j.1469-8137.2001.00034.x, PMID: 33874640

[ref117] Sharon A. Fuchs Y. Anderson J. D. (1993). The elicitation of ethylene biosynthesis by a *Trichoderma xylanase* is not related to the cell wall degradation activity of the enzyme. Plant Physiol. 102, 1325–1329. doi: 10.1104/pp.102.4.1325, PMID: 12231909 PMC158923

[ref118] Shimizu T. Nakano T. Takamizawa D. Desaki Y. Ishii-Minami N. Nishizawa Y.. (2010). Two LysM receptor molecules, CEBiP and OsCERK1, cooperatively regulate chitin elicitor signaling in rice. Plant J. 64, 204–214. doi: 10.1111/j.1365-313X.2010.04324.x, PMID: 21070404 PMC2996852

[ref119] Snelders N. C. Kettles G. J. Rudd J. J. Thomma B. P. H. J. (2018). Plant pathogen effector proteins as manipulators of host microbiomes? Mol. Plant Pathol. 19, 257–259. doi: 10.1111/mpp.12628, PMID: 29368817 PMC5817402

[ref120] Snelders N. C. Petti G. C. van den Berg G. C. M. Seidl M. F. Thomma B. P. H. J. (2021). An ancient antimicrobial protein co-opted by a fungal plant pathogen for in planta mycobiome manipulation. Proc. Natl. Acad. Sci. 118:e2110968118. doi: 10.1073/pnas.2110968118, PMID: 34853168 PMC8670511

[ref121] Snelders N. C. Rovenich H. Petti G. C. Rocafort M. van den Berg G. C. M. Vorholt J. A.. (2020). Microbiome manipulation by a soil-borne fungal plant pathogen using effector proteins. Nat. Plants 6, 1365–1374. doi: 10.1038/s41477-020-00799-5, PMID: 33139860

[ref122] Steel C. C. Drysdale R. B. (1988). Electrolyte leakage from plant and fungal tissues and disruption of liposome membranes by alpha-tomatine. Phytochemistry 27, 1025–1030. doi: 10.1016/0031-9422(88)80266-8

[ref123] Takeda T. Takahashi M. Nakanishi-Masuno T. Nakano Y. Saitoh H. Hirabuchi A.. (2010). Characterization of endo-1, 3–1, 4-β-glucanases in GH family 12 from *Magnaporthe oryzae*. Appl. Microbiol. Biotechnol. 88, 1113–1123. doi: 10.1007/s00253-010-2781-2, PMID: 20680265

[ref124] Takeda T. Takahashi M. Shimizu M. Sugihara Y. Saitoh H. Fujisaki K. (2022). Apoplastic CBM1-interacting proteins bind conserved carbohydrate-binding module 1 motifs in fungal hydrolases to counter pathogen invasion. bioRxiv [Preprint], 2021.2012.2031.474618. doi: 10.1101/2021.12.31.474618 PMC9521807 36173975

[ref125] Tanaka K. Heil M. (2021). Damage-associated molecular patterns (DAMPs) in plant innate immunity: applying the danger model and evolutionary perspectives. Annu. Rev. Phytopathol. 59, 53–75. doi: 10.1146/annurev-phyto-082718-100146, PMID: 33900789

[ref126] ten Have A. Breuil W. O. Wubben J. P. Visser J. van Kan J. A. (2001). *Botrytis cinerea* endopolygalacturonase genes are differentially expressed in various plant tissues. Fungal Genet. Biol. 33, 97–105. doi: 10.1006/fgbi.2001.1269, PMID: 11456462

[ref127] ten Have A. Mulder W. Visser J. van Kan J. A. (1998). The endopolygalacturonase gene Bcpg1 is required for full virulence of *Botrytis cinerea*. Mol. Plant-Microbe Interact. 11, 1009–1016. doi: 10.1094/mpmi.1998.11.10.1009, PMID: 9768518

[ref128] Thomma B. P. H. J. Nürnberger T. Joosten M. H. A. J. (2011). Of PAMPs and effectors: The blurred PTI-ETI dichotomy. Plant Cell 23, 4–15. doi: 10.1105/tpc.110.082602, PMID: 21278123 PMC3051239

[ref129] Tundo S. Paccanaro M. C. Bigini V. Savatin D. V. Faoro F. Favaron F.. (2021). The *fusarium graminearum* FGSG_03624 xylanase enhances plant immunity and increases resistance against bacterial and fungal pathogens. Int. J. Mol. Sci. 22:10811. doi: 10.3390/ijms221910811, PMID: 34639149 PMC8509205

[ref130] Tundo S. Paccanaro M. C. Elmaghraby I. Moscetti I. D’Ovidio R. Favaron F.. (2020). The xylanase inhibitor TAXI-I increases plant resistance to *Botrytis cinerea* by inhibiting the BcXyn11a xylanase necrotizing activity. Plan. Theory 9:601. doi: 10.3390/plants9050601, PMID: 32397168 PMC7285161

[ref131] Turner E. M. C. (1961). An enzymic basis for pathogenic specificity in *Ophiobolus graminis*. J. Exp. Bot. 12, 169–175. doi: 10.1093/jxb/12.1.169

[ref132] Tzelepis G. Dubey M. Jensen D. F. Karlsson M. (2015). Identifying glycoside hydrolase family 18 genes in the mycoparasitic fungal species *Clonostachys rosea*. Microbiology 161, 1407–1419. doi: 10.1099/mic.0.000096, PMID: 25881898

[ref133] Vaahtera L. Schulz J. Hamann T. (2019). Cell wall integrity maintenance during plant development and interaction with the environment. Nat. Plants 5, 924–932. doi: 10.1038/s41477-019-0502-0, PMID: 31506641

[ref134] van der Burgh A. M. Joosten M. (2019). Plant immunity: thinking outside and inside the box. Trends Plant Sci. 24, 587–601. doi: 10.1016/j.tplants.2019.04.009, PMID: 31171472

[ref135] Veneault-Fourrey C. Commun C. Kohler A. Morin E. Balestrini R. Plett J.. (2014). Genomic and transcriptomic analysis of Laccaria bicolor CAZome reveals insights into polysaccharides remodelling during symbiosis establishment. Fungal Genet. Biol. 72, 168–181. doi: 10.1016/j.fgb.2014.08.007, PMID: 25173823

[ref136] Wang Y. Xu Y. P. Sun Y. J. Wang H. B. Qi J. M. Wan B. W.. (2018). Leucine-rich repeat receptor-like gene screen reveals that *Nicotiana* RXEG1 regulates glycoside hydrolase 12 MAMP detection. Nat. Commun. 9:594. doi: 10.1038/s41467-018-03010-8, PMID: 29426870 PMC5807360

[ref137] Wanke A. Malisic M. Wawra S. Zuccaro A. (2021). Unraveling the sugar code: the role of microbial extracellular glycans in plant-microbe interactions. J. Exp. Bot. 72, 15–35. doi: 10.1093/jxb/eraa414, PMID: 32929496 PMC7816849

[ref138] Wanke A. Rovenich H. Schwanke F. Velte S. Becker S. Hehemann J.-H.. (2020). Plant species-specific recognition of long and short β-1,3-linked glucans is mediated by different receptor systems. Plant J. 102, 1142–1156. doi: 10.1111/tpj.14688, PMID: 31925978

[ref139] Wubben J. Mulder W. Ten Have A. Van Kan J. Visser J. (1999). Cloning and partial characterization of endopolygalacturonase genes from *Botrytis cinerea*. Appl. Environ. Microbiol. 65, 1596–1602. doi: 10.1128/AEM.65.4.1596-1602.1999, PMID: 10103256 PMC91226

[ref140] Xia Y. Ma Z. Qiu M. Guo B. Zhang Q. Jiang H.. (2020). N-glycosylation shields *Phytophthora sojae* apoplastic effector PsXEG1 from a specific host aspartic protease. Proc. Natl. Acad. Sci. 117, 27685–27693. doi: 10.1073/pnas.2012149117, PMID: 33082226 PMC7959567

[ref141] Yang C. Liu R. Pang J. Ren B. Zhou H. Wang G.. (2021). Poaceae-specific cell wall-derived oligosaccharides activate plant immunity via OsCERK1 during *Magnaporthe oryzae* infection in rice. Nat. Commun. 12, 2178–2178. doi: 10.1038/s41467-021-22456-x, PMID: 33846336 PMC8042013

[ref142] Yang C. Yu Y. Huang J. Meng F. Pang J. Zhao Q.. (2019). Binding of the *Magnaporthe oryzae* chitinase MoChia1 by a rice tetratricopeptide repeat protein allows free chitin to trigger immune responses. Plant Cell 31, 172–188. doi: 10.1105/tpc.18.00382, PMID: 30610168 PMC6391695

[ref143] Yang Y. Zhang Y. Li B. Yang X. Dong Y. Qiu D. (2018). A *Verticillium dahliae* pectate lyase induces plant immune responses and contributes to virulence. Front. Plant Sci. 9:1271. doi: 10.3389/fpls.2018.01271, PMID: 30271415 PMC6146025

[ref144] Yano A. Suzuki K. Uchimiya H. Shinshi H. (1998). Induction of hypersensitive cell death by a fungal protein in cultures of tobacco cells. Mol. Plant-Microbe Interact. 11, 115–123. doi: 10.1094/mpmi.1998.11.2.115

[ref145] Yin Z. Wang N. Pi L. Li L. Duan W. Wang X.. (2021). *Nicotiana benthamiana* LRR-RLP NbEIX2 mediates the perception of an EIX-like protein from *Verticillium dahliae*. J. Integr. Plant Biol. 63, 949–960. doi: 10.1111/jipb.13031, PMID: 33205907

[ref146] Zerillo M. M. Adhikari B. N. Hamilton J. P. Buell C. R. Lévesque C. A. Tisserat N. (2013). Carbohydrate-active enzymes in *Pythium* and their role in plant cell wall and storage polysaccharide degradation. PLoS One 8:e72572. doi: 10.1371/journal.pone.0072572, PMID: 24069150 PMC3772060

[ref147] Zhang B. Gao Y. Zhang L. Zhou Y. (2021a). The plant cell wall: biosynthesis, construction, and functions. J. Integr. Plant Biol. 63, 251–272. doi: 10.1111/jipb.13055, PMID: 33325153

[ref148] Zhang L. Hua C. Pruitt R. N. Qin S. Wang L. Albert I.. (2021b). Distinct immune sensor systems for fungal endopolygalacturonases in closely related Brassicaceae. Nat. Plants 7, 1254–1263. doi: 10.1038/s41477-021-00982-2, PMID: 34326531

[ref149] Zhang L. Kars I. Essenstam B. Liebrand T. W. Wagemakers L. Elberse J.. (2014). Fungal endopolygalacturonases are recognized as microbe-associated molecular patterns by the *arabidopsis* receptor-like protein RESPONSIVENESS TO BOTRYTIS POLYGALACTURONASES1. Plant Physiol. 164, 352–364. doi: 10.1104/pp.113.230698, PMID: 24259685 PMC3875813

[ref150] Zhang F. Labourel A. Haon M. Kemppainen M. Da Silva Machado E. Brouilly N.. (2021c). The ectomycorrhizal basidiomycete *Laccaria bicolor* releases a GH28 polygalacturonase that plays a key role in symbiosis establishment. New Phytol. 233, 2534–2547. doi: 10.1111/nph.17940 34942023

[ref151] Zhang L. Yan J. Fu Z. Shi W. Ninkuu V. Li G.. (2021d). FoEG1, a secreted glycoside hydrolase family 12 protein from fusarium oxysporum, triggers cell death and modulates plant immunity. Mol. Plant Pathol. 22, 522–538. doi: 10.1111/mpp.13041, PMID: 33675158 PMC8035634

[ref152] Zhao Z. Liu H. Wang C. Xu J. R. (2013). Comparative analysis of fungal genomes reveals different plant cell wall degrading capacity in fungi. BMC Genomics 14:274. doi: 10.1186/1471-2164-14-274, PMID: 23617724 PMC3652786

[ref153] Zhu W. Ronen M. Gur Y. Minz-Dub A. Masrati G. Ben-Tal N.. (2017). BcXYG1, a secreted xyloglucanase from *Botrytis cinerea*, triggers both cell death and plant immune responses. Plant Physiol. 175, 438–456. doi: 10.1104/pp.17.00375, PMID: 28710128 PMC5580746

