# Supplementary material for: Secreted Glycoside Hydrolase Proteins as Effectors and Invasion Patterns of Plant-Associated Fungi and Oomycetes
Source: Front Plant Sci. 2022 Mar 10;13:853106. doi: 10.3389/fpls.2022.853106 (PMC8960721; doi:10.3389/fpls.2022.853106)
Supplement: Supplementary file 1 [file Table_1.docx]

**Supplementary Table 1.** **Selected secreted glycoside hydrolase (GH) proteins from plant-associated fungi and oomycetes with roles in promoting plant colonization and/or activating plant immune responses.**

| **Protein name** | **GH family classification** | **Plant-associated fungus or oomycete (and lifestyle)** | **Characterized role in promoting host colonization and/or activating the plant immune system*** | **Corresponding PRR if known (and plant)** | **References** |
| --- | --- | --- | --- | --- | --- |
| Avenacinase | GH3 | *Gaeumannomyces graminis* var. *avenae* (necrotrophic pathogen) | Avenacinase. Detoxifies the antifungal saponin avenacin in oat [7]. Pathogenicity factor in oat. |  | Turner (1961); Osbourn et al. (1991); Bowyer et al. (1995) |
| Tomatinase | GH3 | *Septoria lycopersici* (necrotrophic pathogen) | Tomatinase. Detoxifies the antifungal saponin α-tomatine to β2-tomatine in tomato [7]. Pathogenicity factor in tomato. |  | Sandrock et al. (1995); Bouarab et al. (2002) |
| GH7.188 | GH5 | *Lasiodiplodia theobromae* (necrotrophic pathogen) | Putative cellulase. Over-expression increases pathogen virulence in grape. |  | Yan et al. (2018) |
| LbGH5-CBM1 | GH5 | *Laccaria bicolor* (ectomycorrhizal fungus) | Involved in the establishment of symbiosis in poplar. |  | Zhang et al. (2018), Zhang et al. (2021b) |
| PsGH7a | GH7 | *Phytophthora sojae* (hemibiotrophic pathogen) | Cellobiohydrolase. Triggers cell death in diverse plant species. Virulence factor. Requires enzymatic activity to trigger cell death and for virulence function (*Nicotiana benthamiana*). |  | Tan et al. (2020) |
| FoTom1 | GH10 | *Fusarium oxysporum* f. sp. *lycopersici* (hemibiotrophic pathogen) | Tomatinase. Detoxifies the antifungal saponin α-tomatine to tomatidine in tomato [7]. Virulence factor in tomato. |  | Roldan-Arjona et al. (1999), Pareja-Jaime et al. (2008) |
| CfTom1 | GH10 | *Cladosporium* *fulvum* (biotrophic pathogen) | Tomatinase. Detoxifies the antifungal saponin α-tomatine to tomatidine in tomato [7]. Virulence factor in tomato. |  | Ökmen et al. (2013) |
| FGSG_11487  FGSG_11304 | GH10 | *Fusarium graminearum* (hemibiotrophic pathogen) | Xylanases. FGSG_11304: Enzymatic activity inhibited by wheat xylanase inhibitor XIP1.  FGSG_11487: Triggers cell death in wheat independently of enzymatic activity. Cell death, but not enzymatic activity, inhibited by XIP1. |  | Tundo et al. (2015) |
| RSAG8_07159 | GH10 | *Rhizoctonia solani* (necrotrophic pathogen) | Triggers cell death in *N. benthamiana*. |  | Anderson et al. (2017) |
| FgXyr1 and FgPg1 | GH10 and GH28 | *F. graminearum* (hemibiotrophic pathogen) | Xylanase and polygalacturonase, respectively. Function synergistically to promote virulence in soybean and wheat. Individually not important for virulence. |  | Paccanaro et al. (2017) |
| VmXyl1 | GH10 | *Valsa mali* (necrotrophic pathogen) | Xylanase. Virulence factor in apple. |  | Yu et al. (2018) |
| PpXyn1 and PpXyn2 | GH10 | *Phytophthora parasitica* (hemibiotrophic pathogen) | Xylanases. Virulence factors in *Nicotiana benthamiana* and tomato. |  | Lai & Liou (2018) |
| RcXYN1 | GH10 | *Rhizoctonia cerealis* (necrotrophic pathogen) | Xylanase. Triggers cell death in wheat and *N. benthamiana*. Virulence factor in wheat. |  | Lu et al. (2020) |
| Xyn1 and Xyn2 | GH10 | *Ustilago maydis* | Xylanases. Virulence factors in maize. |  | Moreno-Sánchez et al. (2021) |
| TvEIX | GH11 | *Trichoderma viride* (root symbiont and biocontrol agent) | β-1-4-endoglucanase (xylanase). Recognised as a MAMP in *N. benthamiana* cv. Xanthi and tomato, triggering cell death and other immune responses. | LRR-RLPs SlEIX1 and SlEIX2 (tomato). Unknown (*N. benthamiana* cv. Xanthi) | Bailey et al. (1990); Furman Matarasso et al. (1999); Ron and Avni (2004) |
| Xylanase 11 | GH11 | *Trichoderma reesei* (root symbiont and biocontrol agent) | Xylanase. Triggers cell death and other immune responses in *Nicotiana tabacum* and tomato independent of enzymatic activity. |  | Enkerli et al. (1999) |
| BcXyn11A | GH11 | *Botrytis cinerea* (necrotrophic pathogen) | β-1,4-endoxylanase. Recognized as a MAMP in tomato and *N. tabacum*, triggering cell death and other immune responses [2]. Virulence factor in tomato. |  | Brito et al. (2006), Frías et al. (2019), Noda et al. (2010) |
| FG_03624  FGSG_10999 | GH11 | *F. graminearum* (hemibiotrophic pathogen) | Xylanases. Triggers cell death in wheat. Enzymatic and cell death activity inhibited by *Triticum aestivum* xylanase inhibitor TAXI III. Cell death activity independent of enzymatic activity. |  | Sella et al. (2013), Tundo et al. (2015), Moscetti et al. (2015), Tundo et al. (2021) |
| VdEIX3 | GH11 | *Verticillium dahliae* (hemibiotrophic pathogen) | Recognised as a MAMP in *N. benthamiana*, triggering cell death and other immune responses [2]. | LRR-RLP NbEIX2 (*N. benthamiana*) | Yin et al. (2021) |
| Vd424Y | GH11 | *V. dahliae* (hemibiotrophic pathogen) | Virulence factor. Possibly localises to plant nucleus [8]. Triggers cell death in several plant species. Cell death dependent on BAK1 and SOBIR1 in *N. benthamiana*. |  | Liu et al. (2021) |
| Xyn11A | GH11 | *U. maydis* | Xylanase. Virulence factor in maize. |  | Moreno-Sánchez et al. (2021) |
| PsXEG1 | GH12 | *P. sojae* (hemibiotrophic pathogen) | Xyloglucanase. Recognized as a MAMP in *N. benthamiana* [2] . Triggers cell death in other solanaceous plants, including *N. benthamiana*. Virulence factor in soybean. Interacts with soybean glucanase inhibitor protein GmGIP1. | LRR-RLP XEG1 (*N. benthamiana*) | Ma et al. (2015b); Ma et al. (2017); Wang et al. (2018) |
| PsXLP1 | GH12 | *P. sojae* (hemibiotrophic pathogen) | Decoy protein for soybean glucanase inhibitor protein GmGIP1 [6]. Virulence factor in the presence of PsXEG1. |  | Ma et al. (2015b); Ma et al. (2017) |
| PpXEG1 | GH12 | *P. parasitica* (hemibiotrophic pathogen) | Xyloglucanase. Interacts with glucanase inhibitor protein NbGIP1 in *N. benthamiana*. |  | Ma et al. (2015b); Ma et al. (2017) |
| PpXLP1 | GH12 | *P. parasitica* (hemibiotrophic pathogen) | Lacks enzyme activity. Triggers cell death in *N. benthamiana*. Interacts with NbGIP1 in *N. benthamiana*. |  | Ma et al. (2015b); Ma et al. (2017) |
| Ps119627  Ps138787  Ps109280 | GH12 | *P. sojae* (hemibiotrophic pathogen) | Trigger cell death in *N. benthamiana.* |  | Ma et al. (2015b) |
| PPTG_16567  PPTG_16566  PPTG_19220  PPTG_16273  PPTG_16272  PPTG_16267  PPTG_16271 | GH12 | *P. parasitica* (hemibiotrophic pathogen) | Trigger cell death in *N. benthamiana.* |  | Ma et al. (2015b) |
| Pc111741  Pc99469  Pc106898  Pc107122  Pc15454  Pc15453 | GH12 | *Phytophthora capsici* (hemibiotrophic pathogen) | Trigger cell death in *N. benthamiana.* |  | Ma et al. (2015b) |
| PITG_06962 | GH12 | *Phytophthora infestans* (hemibiotrophic pathogen) | Triggers cell death in *N. benthamiana*. |  | Ma et al. (2015b) |
| FGSG_05851 | GH12 | *F. graminearum* (hemibiotrophic pathogen) | Triggers cell death in *N. benthamiana.* |  | Ma et al. (2015b) |
| VdEG1  VdEG3 | GH12 | *V. dahliae* (hemibiotrophic pathogen) | Triggers cell death independent of enzyme activity in *N. benthamiana*. Virulence factor in cotton. Enzymatic activity required for virulence. |  | Gui et al. (2017) |
| BcXyg1 | GH12 | *B. cinerea* (necrotrophic pathogen) | Triggers cell death in dicot plants independent of enzyme activity. |  | Zhu et al. (2017) |
| FoEG1 | GH12 | *F. oxysporum* (hemibiotrophic pathogen) | Recognized as a MAMP in *N. tabacum*, *N. benthamiana*, tomato and cotton, triggering cell death and other immune responses [2]. Virulence factor in cotton. |  | Zhang et al. (2021d) |
| MoCel12A and MoCel12B | GH12 | *Magnaporthe oryzae* (hemibiotrophic pathogen) | β-glucanase (endoglucanase). Releases oligosaccharides from hemicellulose in rice cell walls; predicted to support nutrition for fungal growth [3]. | OsCERK1-OsCEBiP complex (rice) | Yang et al. (2021) |
| BcGs1 | GH15 | *B. cinerea* (necrotrophic pathogen) | Triggers cell death in tomato, cucumber, pea and *N. tabacum*. Induces expression of plant defence response genes and lignin accumulation in tomato. |  | Zhang et al. (2015); Yang et al. (2018a) |
| OPEL | GH16 | *P. parasitica* (hemibiotrophic pathogen) | Cell death elicitor, possibly due to DAMP release [4]. Induces expression of plant defence genes. |  | Chang et al. (2015) |
| BcCrh1 | GH16 | *B. cinerea* (necrotrophic pathogen) | Transglycosylase. Cytoplasmic effector involved in fungal cell wall biosynthesis. Triggers cell death and other defence responses in *N. benthamiana* and tomato. |  | Bi et al. (2021) |
| CfGH17-1  CfGH17-5 | GH17 | *Cladosporium fulvum* (biotrophic pathogen) | CfGH17-1: 1,3-β-glucanase Releases (oligo)saccharides from tomato cell wall (predicted to support fungal growth and reproduction through nutrition) [3]. Released (oligo)saccharides trigger cell death upon recognition as DAMPs in tomato [4].  CfGH17-5: Predicted 1,3-β-glucanase.Triggers cell death in tomato. |  | Ökmen et al. (2019) |
| DsGH17-1 | GH17 | *Dothistroma septosporum* (hemibiotrophic pathogen) | Predicted 1,3-β-glucanase. Triggers cell death in tomato. |  | Ökmen et al. (2019) |
| MfGH17-1 | GH17 | *Pseudocercospora fijiensis* (hemibiotrophic pathogen) | Predicted 1,3-β-glucanase. Triggers cell death in specific cultivars of tomato. |  | Ökmen et al. (2019) |
| VDECH | GH18 | *V. dahliae* (hemibiotrophic pathogen) | Triggers cell death and induction of defence genes in *Arabidopsis* and cotton. |  | Cheng et al. (2017) |
| MpChi | GH18 | *Moniliophthora pernicosa* (hemibiotrophic pathogen) | Inactive chitinase. Sequesters chitin fragments to prevent chitin-triggered immunity [5A]. |  | Fiorin et al. (2018) |
| MrChi | GH18 | *Moniliophthora rorei* (hemibiotrophic pathogen) | Inactive chitinase. Sequesters chitin fragments to prevent chitin-triggered immunity [5A]. |  | Fiorin et al. (2018) |
| MoChia1/MoChi1 | GH18 | *M. oryzae* (hemibiotrophic pathogen) | Chitinase. Triggers cell death independently of enzymatic activity in rice. Virulence factor. Sequesters chitin fragments to prevent chitin-triggered immunity in rice [5A]. Interacts with OsMBL1 in rice. | Tetratricopeptide-repeat protein OsTPR1 (rice) | Han et al. (2019); Yang et al. (2019) |
| UvCBP1 | GH18 | *Ustilaginoidea virens* (biotrophic pathogen) | Virulence factor. Competes with rice OsCEBiP for the binding of chitin (i.e. may sequester chitin fragments to suppress chitin-triggered immunity [5A]). |  | Li et al. (2021b) |
| MbA_GH25 | GH25 | *Moesziomyces bullatus* ex *Albugo* (epiphytic yeast) | Lysozyme. Reduces infection by the oomycete *Albugo laibachii* (white rust disease pathogen) in *Arabidopsis thaliana* at the phyllosphere. |  | Eitzen et al. (2021) |
| BcPG1  BcPG2  BcPG3  BcPG4  BcPG5  BcPG6 | GH28 | *B. cinerea* (necrotrophic pathogen) | Endopolygalacturonases. Trigger cell death or chlorosis on a number of plant species independently (BcPG1, BcPG3) or dependently (BcPG2) of enzymatic activity. Virulence factors. BcPG2 is inhibited by polygalacturonase inhibitor protein VvPGIP1 of grape. | LRR-RLP AtRBPG1/RBPG1 (*A. thaliana*) | ten Have et al. (1998); Wubben et al. (1999); Kars et al. (2005); Joubert et al. (2007); Zhang et al. (2014) |
| AcPG1 | GH28 | *Alternaria citri* (necrotrophic pathogen) | Endopolygalacturonase. Virulence factor on citrus fruit. |  | Isshiki et al. (2001) |
| CLPG1 | GH28 | *Colletotrichum lindemuthianum* (hemibiotrophic pathogen) | Endopolygalacturonase. Triggers plant cell death and induces plant defence response in *N. tabacum* dependent on enzyme activity. |  | Boudart et al. (2003) |
| LbGH28A | GH28 | *L. bicolor* (ectomycorrhizal fungus) | Endopolygalacturonase. Involved in the establishment of symbiosis. |  | Zhang et al. (2021b) |
| BcAra1 | GH43 | *B. cinerea* (necrotrophic pathogen) | Endoarabinanase. Host-specific contribution to virulence. |  | Nafisi et al. (2014) |
| MoAbfB | GH43 | *M. oryzae* (hemibiotrophic pathogen) | α-L-arabinofuranosidase. Required for full virulence in rice. Degrades the rice cell wall, releasing oligosaccharides that are recognized as DAMPs [4]. |  | Wu et al. (2016) |
| EG1 | GH45 | *R. solani* (necrotrophic pathogen) | Endoglucanohydrolase (cellulase). Recognized as a MAMP in maize, *N. tabacum* and *A. thaliana* [2], triggering cell death and other immune responses. |  | Guo et al. (2021); Ma et al. (2015a) |
| Um01829/UmAfg1/UmErc1 | GH51 | *U. maydis* (biotrophic pathogen) | Exo-1,3-β-glucanase. Organ-specific virulence factor. Sequesters 1,3-β-glucan to prevent ROS burst [5A]. Involved in fungal cell-to-cell movement in maize bundle sheath cells. |  | Lanver et al. (2014); Schilling et al. (2014); Ökmen et al. (in preparation) |
| UhErc1 | GH51 | *U. hordei* | Exo-1,3-β-glucanase. Organ-specific virulence factor. Sequesters 1,3-β-glucan to prevent ROS burst [5A]. Involved in fungal cell-to-cell movement in maize bundle sheath cells. |  | Ökmen et al. (in preparation) |
| Ssaxp | GH54 | *S. sclerotiorum* (necrotrophic pathogen) | Putative arabinofuranosidase/β-xylosidase. Virulence factor in Canola. |  | Yajima et al. (2009) |
| Arb93B | GH93 | *F. graminearum* (hemibiotrophic pathogen) | Predicted arabinanase. Virulence factor in wheat. Suppresses plant immune responses. |  | Hao et al. (2019) |

DAMP, damage-associated molecular pattern; MAMP, microbe-associated molecular pattern; GH, glycoside hydrolase; LRR, leucine-rich repeat; PRR, pattern recognition receptor; RLP, receptor-like protein; ROS, reactive oxygen species. *Corresponding panel number in Figure 1, where known, is shown in square brackets.
